# Independent recruitment of *Igh* alleles in V(D)J recombination

**DOI:** 10.1038/ncomms6623

**Published:** 2014-12-17

**Authors:** Clara F. Alves-Pereira, Raquel de Freitas, Telma Lopes, Rui Gardner, Filipa Marta, Paulo Vieira, Vasco M. Barreto

**Affiliations:** 1 Epigenetics and Soma Laboratory, Instituto Gulbenkian de Ciência, Rua da Quinta Grande, n° 6, 2780-156 Oeiras, Portugal; 2 Flow Cytometry Laboratory UIC, Instituto Gulbenkian de Ciência, Rua da Quinta Grande, n° 6, 2780-156 Oeiras, Portugal; 3 Unité Lymphopoïèse, Institut Pasteur, 25, Rue du Dr Roux, 75724 Paris, France; 4 INSERM U668, F-75015 Paris, France

## Abstract

How the vast majority of B cells express only one of the two alleles at their immunoglobulin loci remains a biological puzzle. Here, in mice reconstituted with a single haematopoietic stem cell, we demonstrate that each of the two immunoglobulin heavy chain (*Igh*) alleles has a similar probability to be the first to undergo *V*_*H*_ to *DJ*_*H*_ rearrangement. We also observe this similar probability in clones from multipotent and common lymphoid precursors. The extreme biases in the expression of the alleles that we find in more differentiated subsets are mostly due to constraints imposed by early rearrangements. Our data demonstrate that each of the two *Igh* alleles in a B cell behaves independently of the other, up to the moment when a successful rearrangement in one allele triggers a feedback mechanism that prevents further recombination.

## Abstract (web summary)

B cells rearrange the immunoglobulin heavy chain genes to produce a functional B cell receptor, but how it is decided that one allele rearranges first is not clear. Here the authors provide evidence that in the majority of common lymphoid precursor clones, the two alleles have a similar probability of rearranging first.

## Graphical abstract

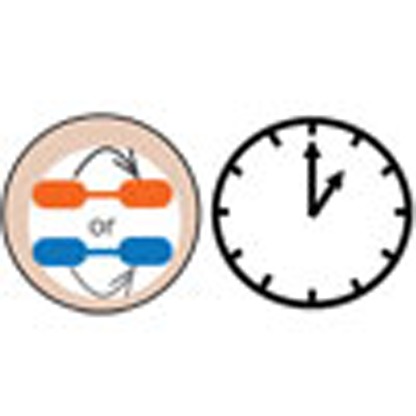

Each B cell expresses immunoglobulin heavy chains at its surface, typically encoded by only one of the two alleles^1^. The question of why this phenomenon of allelic exclusion occurs has generated a number of non-exclusive and untested answers. For instance, it could be a solution to produce one or a limited set of antigen receptors per cell, as postulated by the clonal selection theory^2^, or a safety mechanism to decrease the chances of detrimental translocations^3^, since antigen receptor genes undergo somatic recombination^4^. How allelic exclusion arises would seem to be more tractable; however, this is an ongoing question since the 1960s. Mostly because of the imprecise junction of the *V*_*H*_, *D*_*H*_ and *J*_*H*_ gene segments that are found in the immunoglobulin heavy chain (*Igh*) locus, only a minor proportion of the rearrangements will be ‘productive’ or *V*_*H*_*DJ*_*H*_^+^ (encoding a full-length chain), which intrinsically ensures some level of allelic exclusion. However, for the murine *Igh* locus, a minimal stochastic model of allelic exclusion based exclusively on the outcome of the rearrangement process^5^ is insufficient to account for the degree of exclusion observed at the surface of the cells^6^.

The *D*_*H*_ to *J*_*H*_ rearrangement step occurs in both *Igh* alleles^7^. This event has recently been proposed to mark the *DJ*_*H*_ junction for secondary rearrangement, that is, the *V*_*H*_ to *DJ*_*H*_ stage^8^, and once a productive secondary rearrangement takes place in one of the alleles of the pro-B cell, expression of the pre-B-cell receptor (pre-BCR) at the surface signals a series of events leading to the shutdown of recombination at the second allele. This feedback mechanism, first suggested for the immunoglobulin kappa locus^9^, and subsequently elaborated for the *Igh* locus^10^, has been independently confirmed in a number of ways. Notably, a μH chain encoded by a transgene or a knocked-in allele inhibits *V*_*H*_ to *DJ*_*H*_ rearrangement at the endogenous *Igh* locus only if expressed at the surface, as part of the pre-BCR^11^,^12^,^13^. In addition, in cells with genotypic inclusion (with both alleles rearranged productively) only one of the chains pairs efficiently with the surrogate light chain^14^ that also forms the pre-BCR and the frequency of cells with genotypic inclusion (*V*_*H*_*DJ*_*H*_^*+*^/*V*_*H*_*DJ*_*H*_^+^) increases in the absence of the surrogate light chain^15^ or when the signalling from an intact pre-BCR is perturbed by mutations in one of its downstream targets^16^. More recently, two additional feedback mechanisms have been proposed, one triggered by an Ataxia Telangiectasia Mutated (ATM)-dependent sensing of the rearrangement at the first *Igh* allele^3^ and the other by a stable μH mRNA with a premature stop codon that cannot be translated into a μH chain^17^. Overall, feedback inhibition and the rearrangement of the second allele in its absence largely explain the proportion of *V*_*H*_*DJ*_*H*_^+^/*DJ*_*H*_ and *V*_*H*_*DJ*_*H*_^+^/*V*_*H*_*DJ*_*H*_^+/−^ IgM^+^ B cells^18^.

The events initially leading to *V*_*H*_ to *DJ*_*H*_ rearrangement that render the *V*_*H*_ regions ‘accessible’ for rearrangement include the relocalization of the *Igh* locus away from the nuclear periphery in pro-B cells^19^ and long-range interactions mediated by looping of the *Igh* domains^20^,^21^ that depend on regulatory regions, namely the one located in the *V*_*H*_ to *D*_*H*_ intergenic region^22^. However, it is unclear whether a specific mechanism ensures that each cell only tests one *V*_*H*_ to *DJ*_*H*_ rearrangement at a time. The acquisition of competence to undergo rearrangement by the *Igh* locus in the course of B-cell differentiation has been described at the molecular level (for example, ref. 23); however, typically these studies are carried out in bulk cultures, averaging out any existing differences between the alleles at the single-cell level. In turn, imaging-assisted studies (for example, refs 19, 20, 21, 24) can discriminate between the alleles at the single-cell level, but measure features that are only correlates of *V*_*H*_*(D*_*H*_*)J*_*H*_ accessibility, which are not always confirmed by different techniques^25^, and provide mere snapshots of a process that is essentially dynamic.

Here we performed a clonal analysis of *Igh* allelic exclusion, and provide conclusive evidence that the allelic exclusion of immunoglobulin receptor loci differs from X-chromosome inactivation, as no stable epigenetic mark is propagated until pro-B cells start rearranging. More importantly, we also show that the *Igh* alleles rearrange independently from each other in the majority of clones derived from common lymphoid precursors.

## Results

### Clonal analysis tracks V(D)J recombination

**Figure 1:**
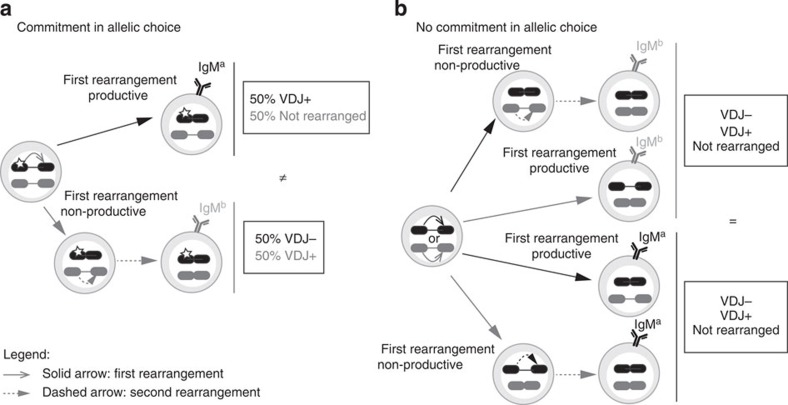
Rationale. The commitment and no commitment models of *Igh* allelic choice predict a distinctive *V*_H_ to *DJ*_H_ rearrangement status in the two IgM^a^ and IgM^b^ fractions of B-cell clonal populations. (**a**) In a B-cell clone that is pre-determined to rearrange first a given allele (represented by a black locus with a white star), all the cells rearrange the marked allele first and the second allele (grey locus) remains non-rearranged, unless the previous rearrangement is non-productive (*V*_H_*DJ*_H_^−^). The resulting clone contains two populations of B cells: one that expresses the product of the first rearrangement (a productive rearrangement, *V*_H_*DJ*_H_^+^) and retains 50% of the alleles non-rearranged (IgM^a^ population in the scheme) and another that expresses the product of the second rearrangement (IgM^b^ population in the scheme) and has both alleles rearranged. (**b**) The alternative model (no commitment) assumes that both alleles are equally accessible, each with equal chance of rearranging first. This condition renders the IgM^a^ and IgM^b^ populations of a cell clone undistinguishable at the structure of the silent *Igh* allele, since both populations will have a similar frequency of silent alleles in the *V*_*H*_*DJ*_*H*_ and *DJ*_*H*_ configurations. The present scheme assumes the ordered model of V(D)J rearrangement^7^, and represents the *V*_*H*_ to *DJ*_*H*_ rearrangement only (the D-to-J rearrangement is biallelic and has occurred before the developmental stage illustrated). In a sorted population of IgM^+^ cells, the frequency of non-rearranged alleles can be measured by quantifying the *V*_*H*_*–D*_*H*_ intergenic fragment retention within the *Igh* locus.

Clonal analysis is typically used to investigate the commitment of precursor cells to a given lineage or set of lineages. We have adapted this technique to test whether an *Igh* allele from a precursor cell is committed to recombine a *V*_*H*_ segment before the other allele as the cell divides and enters the lymphoid lineage. As shown in Fig. 1, although in a bulk culture it is not possible to conclude whether the alleles from cells heterozygous for the *Igh* locus (*Igh*^*a/b*^) are differently committed for rearrangement, in isolated clones the commitment and non-commitment scenarios produce sharply different outcomes. In the case of commitment, all the cells expressing the μ chain from the first allele to rearrange (in Fig. 1a, IgM^a^) would have the second allele in the non-*V*_*H*_-rearranged *DJ*_*H*_ configuration and the remaining IgM^+^ cells (in Fig. 1a, IgM^b^) would have the second allele in the *V*_*H*_*DJ*_*H*_ configuration. In contrast, if commitment does not occur (Fig. 1b), the IgM^a^ and IgM^b^ pools of cells would not differ in the status of rearrangement of the silenced allele, since each pool would have the same proportion of IgM^+^ cells with a unique *V*_*H*_ to *DJ*_*H*_ rearrangement versus cells that were rescued by the second *V*_*H*_ to *DJ*_*H*_ rearrangement. Thus, *D*_*H*_ to *J*_*H*_ and *V*_*H*_ to *DJ*_*H*_ junctions function as a natural reporter system, since the signature junction sequences somatically introduced in the *Igh* locus help to ascertain when rearrangement occurred in each clone as the cells divide.

### Both *Igh* alleles can rearrange first in clones derived from HSC

The main feature of the *Igh* locus and other antigen receptor loci is V(D)J recombination; however, these genes are frequently included in a growing group of autosomal genes displaying what has been called random monoallelic expression^26^. Unlike loci under parental origin-specific imprinting, in these genes roughly 50% of the cells express the maternal allele and the remaining 50% express the paternal allele. Since the parallel with X-chromosome inactivation has been made in several reports^27^,^28^,^29^ and haematopoietic stem cells (HSCs) show X-chromosome inactivation, we first asked whether in mice with single-HSC-derived haematopoietic systems, the emerging IgM^a^ and IgM^b^ populations would also show signs of a commitment that is already present in the HSC and that is then stably propagated.

Cells heterozygous for a pan-leukocitary marker polymorphism (CD45.1/45.2), the *Igh* allotypes (*Igh*^a/b^) and the X-linked gene *Foxp3* and its *gfp*-fused knock-in (*Foxp*3^gfp/wt^) were single-cell-sorted from the HSC-long-term repopulation potential-enriched Lin^−^Rho^−^CD45^int^Hoe^−^ side population (SP) pool of C57BL/6J female bone marrows (BMs; see Methods; Supplementary Fig. 1). Sublethally irradiated B6-*Rag2*^*−/−*^ was then single-cell-reconstituted and monitored over time to detect animals showing chimerism in the blood. The percentage of reconstituted animals was 4.8–22.9%. This low efficiency is in the range of what has been described^30^, and further increases the likelihood of obtaining a collection mainly composed of animals with monoclonal haematopoietic systems. Animals with chimerism (*N*=15) were euthanized within 8–18 weeks from the single-cell transplant, analysed by flow cytometry for the presence of lymphoid and myeloid cells and in some cases used to produce secondary recipients, attesting to their long-term repopulation potential (Table 1). The collection analysed for allelic exclusion comprised animals that were long-term and short-term reconstituted.

**Figure 2:**
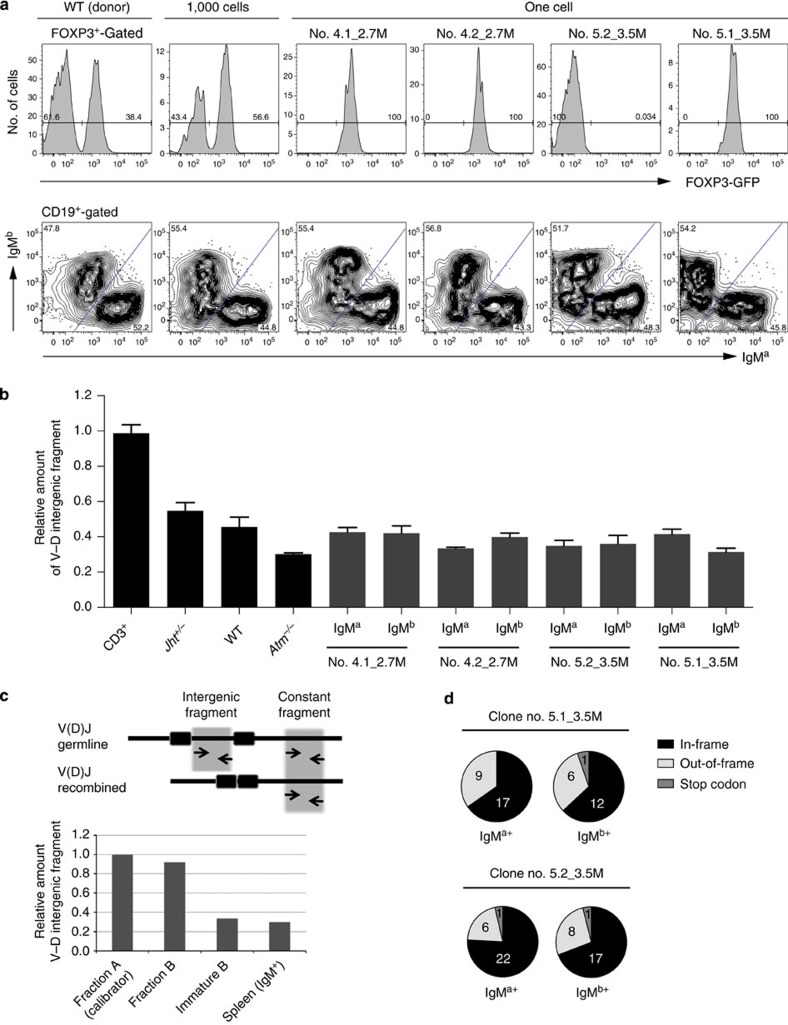
Direct test for a stable *Igh* epigenetic commitment in HSC that dictates which allele undergoes *V*_*H*_ to *DJ*_*H*_ rearrangement first in the B lineage. (**a**) The expression of FOXP3GFP in lymph node (LN)-derived regulatory T cells is bimodal in the non-clonal populations, and unimodal in mice reconstituted with a single HSC (top histograms), but the expression of the *Igh* alleles is balanced in the splenic B cells of both the non-clonal and single-cell-reconstituted mice (bottom contour plots). Top histograms represent GFP expression profiles of CD45.1^+^CD4^+^FOXP3^+^-gated LN cells, and bottom contour plots show the CD45.1^+^CD19^+^-gated splenic B-cell surface IgM^a^/IgM^b^ profiles of the same clonal and non-clonal samples. (**b**) Genomic quantification with qPCR of *in vivo*-differentiated B-cell clones. The retention of the *V*_*H*_–*D*_*H*_ intergenic fragment in IgM^a^ and IgM^b^-sorted splenic populations from each of the single-HSC clones—the same samples shown in **a**—is similar to each other, and in the same range of IgM^+^-sorted cells from non-clonal populations. The qPCR on DNA from IgM^+^ cells purified from *Jht*^*+/−*^ mice (that can only rearrange one of the *Igh* alleles) and *Atm*^*−/−*^ mice (which have a higher frequency of *V*_*H*_*DJ*_*H*_*/V*_*H*_*DJ*_*H*_ cells than the WT^3^,^22^) produced the expected results. Error bars are the s.e.m. of three independent runs of the same samples in different input amounts. (**c**) The reduction in the amount of the *V*_*H*_–*D*_*H*_ intergenic fragment from the fractions A and B of pro-B cells to immature B cells shows that the assay is reporting the changes in the structure of the *Igh* locus throughout development. (**d**) Comparison of the frequency of productive and non-productive *V*_*H*_*DJ*_*H*_ rearrangements detected in the splenic IgM^a^ and IgM^b^-sorted pools from clone no. 5.1_3.5 M (*P*=1.00, Fisher’s exact test) and no.5.2_3.5 M (*P*=0.55, Fisher’s exact test).

Female donor cells heterozygous both for the X-linked *Foxp3* and *Igh* loci were used to compare in the same experiment X-chromosome inactivation with *Igh* allelic exclusion. FOXP3 is a transcription factor expressed by regulatory T cells (Tregs). Owing to X-chromosome inactivation, in heterozygous animals for a knock-in green fluorescent protein (GFP fusion protein; *Foxp3*^*gfp/wt*^)^31^ only 50% of Tregs are green (Fig. 2a). Control animals reconstituted with 1,000 HSC-enriched SP cells reproduced this balanced bimodal distribution of the GFP signal in Tregs (Fig. 2a, Table 1), which became biased when less cells were used in the reconstitution and strictly unimodal (0% GFP or 100% GFP) in monoclonal haematopoietic systems. We confirmed that each individual HSC has one inactivated X-chromosome and that inactivation is a stable epigenetic property, functioning here as an internal control for the monoclonality of the reconstitutions.

We then analysed *Igh* allelic exclusion in the single HSC-reconstituted animals. We consistently found roughly equal numbers of IgM^a^ and IgM^b^ B cells per animal (Fig. 2a and Supplementary Table 2), similar to what is found in polyclonal haematopoietic systems^6^. This observation is in sharp contrast to the X-chromosome inactivation data and suggests the lack of stable allelic commitment in the *Igh*; however, as explained above, the definitive read-out relies on the examination of the rearrangement status of the non-expressed allele. Thus, a quantitative PCR assay was designed to measure the fraction of alleles that retain the *V*_*H*_*–D*_*H*_ intergenic region, which is excised as an episome during *V*_*H*_ to *DJ*_*H*_ recombination and is diluted out as the pre-BCR-expressing cell divides and progresses in development (Fig. 2b). The assay reports the expected differences in the retention of the *V*_*H*_*–D*_*H*_ intergenic fragment in *Rag2*^−/−^ pro-B cells (100%) and splenic B cells from wild-type (WT) animals (roughly 60% of cells in *V*_*H*_*DJ*_*H*_*/DJ*_*H*_ configuration and 40% in the *V*_*H*_*DJ*_*H*_*/V*_*H*_*DJ*_*H*_ configuration^32^, which equals 30% of retention), *Jht*^*+/−*^ IgM+ cells (the knockout allele cannot rearrange and therefore all IgM^+^ cells are in the *V*_*H*_*DJ*_*H*_/germline configuration^33^, which equals 50% intergenic fragment retention) and *Atm*^*−/−*^ (which have an intergenic fragment retention below that of WT animals^3^). If allelic commitment exists before rearrangement, then the isolated IgM^a^ and IgM^b^ subpopulations of a given clone must differ drastically in the amount of the *V*_*H*_*–D*_*H*_ intergenic fragment retained (50 versus 0%); however, we did not detect any difference in the four animals analysed (Fig. 2c). Similarly, if allelic pre-commitment exists, the cells would carry a productive rearrangement in the pre-committed allele (either IgM^a^ or IgM^b^) together with no *V*_*H*_ rearrangement in the other, while the cells expressing the other allele would have roughly the same number of productive and non-productive rearrangements. Since the *V*_*H*_*DJ*_*H*_^+^/*V*_*H*_*DJ*_*H*_^−^ ratio may change depending on the *V*_*H*_ family, we matched the type of families selected for analysis per isolated fraction, focusing on the most prominent families: J558 and 7183. We did not find any striking difference in the frequency of *V*_*H*_*DJ*_*H*_^+^ and *V*_*H*_*DJ*_*H*_^−^ rearrangements of IgM^a^ and IgM^b^-isolated splenic B cells from the monoclonally reconstituted animals (Fig. 2d). Taken together, these data conclusively show that, contrary to X-chromosome inactivation, there is no stable propagation of an allelic commitment to rearrange first the *V*_*H*_ segment in the clones that emerge from a single HSC and differentiate *in vivo* into pro-B cells.

### IgM^a/b^ expression becomes skewed in clones derived from CLP

**Figure 3:**
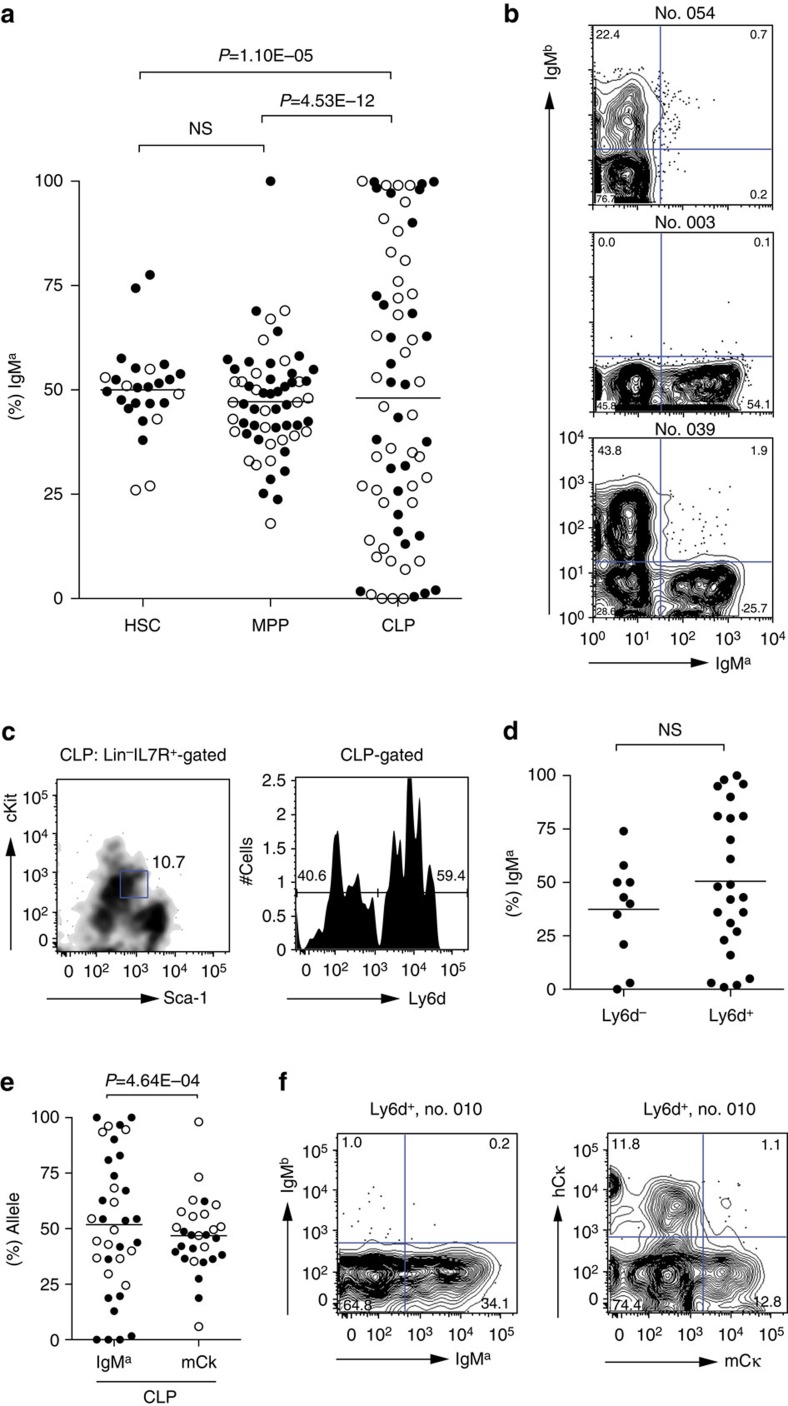
IgM^a/b^ expression in CLP-derived clones. As the developmental stage of the isolated precursor cell approaches the stage when recombination typically occurs, the distribution of IgM^a^/IgM^b^-expressing cells in the resulting B-cell clones differentiated *in vitro* becomes biased. The graphs show *P* values from an F-test. (**a**) IgM^a^ percentage (IgM^a^/[IgM^a^+IgM^b^]) obtained from flow cytometric analysis of B-cell clones differentiated in culture from HSCs, MPPs and CLPs; each dot represents a clone, open and closed dots distinguish two independent assays. (**b**) Representative examples of flow cytometric profiles of CLP-derived clones presenting extreme bias in the expression of IgM^a^ or IgM^b^ (clone no. 054 and no. 003) and a mild biased/balanced (clone no. 039). (**c**) Left: density plot shows the Lin^−^IL7R^+^-gated BM population, staining cKit^int^ and Sca-1^int^, thus identified as the CLP population. Right: histogram shows the Ly6d-staining profile in the CLP-gated population. (**d**) Percentages of IgM^a^-expressing cells in clones derived from CLPs partitioned according to the presence of the Ly6d marker^35^. The percentage of IgM-expressing cell clones emerging per CLP.Ly6d^−^ plated cell was 2%, while Ly6d^+^ cells render IgM^+^ B cell clones at least 10 × more frequently. (**e**) Distribution of the percentages of heavy chain alleles (IgM^a^/ [IgM^a^+IgM^b^]) and light chain alleles (hCκ/[hCκ+mCκ]) in CLP Ly6d^+^-derived clones from the same experiment. (**f**) CLP Ly6d+ clone no. 010 flow cytometric profile for the immunoglobulin heavy chain alleles (IgM^a^/IgM^b^) and light chain alleles (hCκ/mCκ).

We next asked whether *Igh* allelic commitment for *V*_*H*_ to *DJ*_*H*_ rearrangement would emerge at some stage during the lineage differentiation to pro-B cells, by applying the previous clonal approach to progressively more differentiated precursor cells. Given the low expansion potential of more differentiated precursors compared with the HSC, we did not reconstitute mice with single B-cell precursors, and used instead a cell culture system. Single cells from pools enriched for HSCs, multipotent progenitors (MPPs), common lymphoid precursors (CLPs) or Pro-B cells were plated under stimulation conditions that are routinely used to mimic the development that occurs in the BM^34^. As expected, the B-cell-plating efficiency increased from HSCs to CLPs (see Methods; Supplementary Fig. 4), while the time required to detect surface IgM expression decreased. In Fig. 3a, we plot the percentage of IgM^a^-expressing cells (relative to total IgM^+^) per clone growing from seeded HSCs, MPPs and CLPs. As expected, within each group the distribution of IgM^a^- and IgM^b^-expressing cells is similar. However, the dispersion of each of these percentages is much more pronounced in the clones emerging from CLPs than in the clones resulting from the HSCs and the MMP progenitors (CLP versus MPP, *P*=4.5 × 10^−12^ by F-test; CLP versus HSC, *P*=1.1 × 10^−5^ by F-test). Notably, the collection of the CLP-derived clones includes examples of clones completely skewed to IgM^a^ or IgM^b^ (Fig. 3b). Given the heterogeneous nature of the CLP populations, we decided to further dissect them to identify the earliest population containing progenitors with potential for the highly biased expression of IgM^a^ or IgM^b^. The surface marker Ly6d (Fig. 3c) identifies one population (Ly6d^−^) that conserves the full lymphoid potential and one population (Ly6d^+^) that is to date the earliest B-cell progenitor^35^. We could confirm that, under conditions promoting B-cell differentiation, the B-cell-plating efficiency of the Lyd6^−^ CLP-derived clones was at least 10 × lower than in Ly6d^+^ CLP-derived clones (in one experiment, 15 wells presented colonies out of 960 single-cell Lyd6^−^ CLP-plated cells, while 68 wells presented colonies out of 384 single-cell-plated Lyd6^+^ CLP-plated cells). We observed that the highly skewed clones are more frequent in the Ly6d^+^ pool (Fig. 3d), although the difference is not statistically significant. We have further differentiated B-cell clones from whole B220^+^CD43^+^ pro-B cells and from the fraction B and found that, although the lower expansion potential of pro-B cells originated smaller clones, CLP- and B220^+^CD43^+^-derived clones have a similar frequency of skewed clones (Supplementary Fig. 4c). Fraction B presented an even lower expansion potential, which precluded further molecular analysis. We have also compared *Igh* and *Igκ* loci with respect to the allelic bias observed in *in vitro* CLP-derived clones from mice heterozygous for both *Igh* and light chain immunoglobulin loci (B6.Cg-*Igh*^*a/b*^, *hCκ/mCκ*). The *Igh* locus typically rearranges before the kappa light chain and usually four to six cell divisions occur between the rearrangement of these immunoglobulin genes^36^,^37^. As observed in Fig. 3e,f, the bias observed for the *Igh* is not reproduced for the *Igκ* (*P*=0.00046 by F-test) presumably because the number of cells rearranging the *Igκ* alleles independently is much higher than the number of cells rearranging the *Igh* alleles within the clone. The balanced ratio of the Igκ-expressing cells also suggests that the *Igκ* locus is not pre-committed in the CLP stage of B-cell development. Taken together, these data show that both the probability of finding clones with allelic bias and the degree of bias increase as the stage at which the cells are seeded gets closer to the time of V(D)J rearrangement.

### Independent recruitment of *Igh* alleles in CLP-derived clones

**Figure 4:**
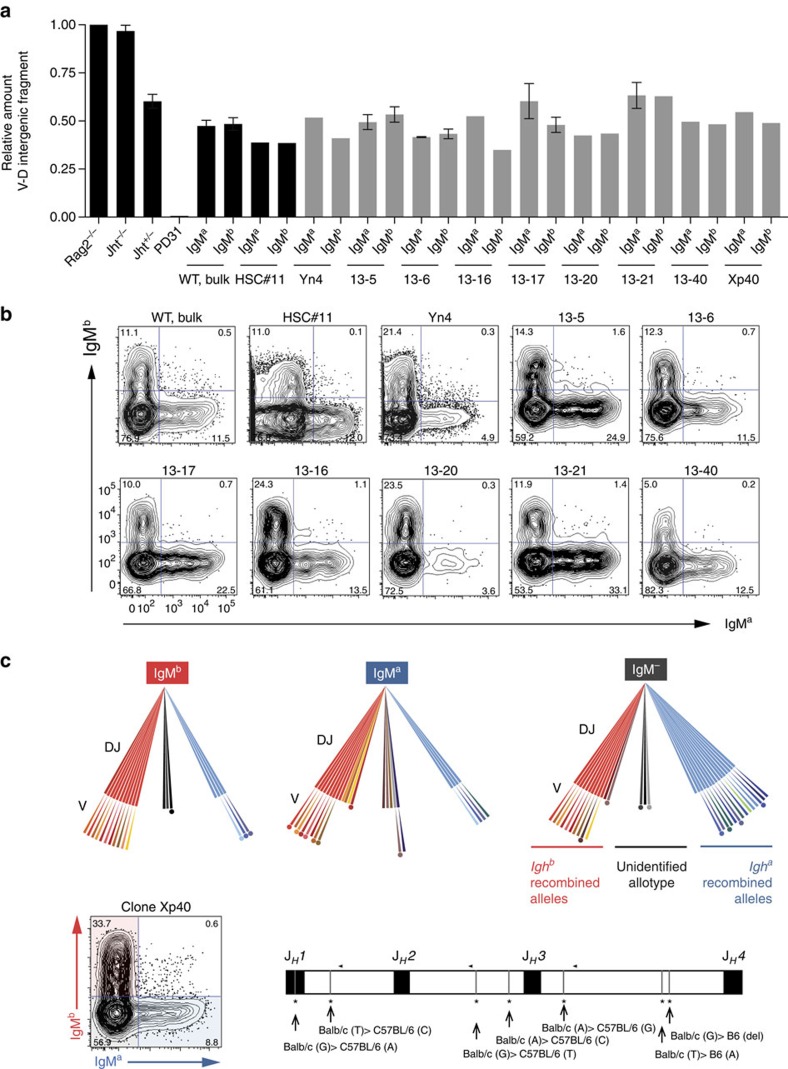
Rearrangement status of the silent *Igh* allele in CLP-derived clones. The status of rearrangement of the silent allele in IgM^a^ and IgM^b^-sorted cells from *in vitro* CLP-derived B-cell clones shows independent allele recruitment for rearrangement. (**a**) *V*_*H*_*–D*_*H*_ intergenic fragment retention quantification data are shown in two blocks: in black, a battery of control profiles: *Rag2*^*−/−*^ and *Jht*^*−/−*^ CD19^+^ cells, that do not rearrange the *Igh* locus, *Jht*^*+/−*^ as in Fig. 2c, the PD31 cell line, which lacks the *V*_*H*_*–D*_*H*_ intergenic fragment^55^, and additional WT controls, all differentiated *in vitro*. In grey, data from pairs of IgM^a^- and IgM^b^-sorted samples from mildly biased/balanced clones. Sample Yn4 is a CLP-Ly6d^−^-derived clone, and the remaining samples are CLP-Ly6d^+^-derived. Error bars denote s.e.m. (**b**) Flow cytometric profiles of the WT bulk control and clones represented in the graph. (**c**) Top panel: schematic representation of the diversity of rearrangements obtained by sequencing *V*_*H*_*DJ*_*H*_ and *DJ*_*H*_ rearrangements of IgM^b^, IgM^a^ and IgM^−^ populations of the clone Xp40 (see Table 2 for a detailed description). Legend: long brush strokes represent D–J joints and short ones the *V*_*H*_*–D*_*H*_ joints. Sequences of *Igh*^a^*, Igh*^*b*^ and unknown allotype are represented by cold, warm and greyish colours, respectively. A long stroke not followed by a short stroke represents a *DJ*_*H*_ rearrangement. A dot represents a non-productive rearrangement. Different colours in longer strokes represent diverse D-to-J joints as different colours in shorter strokes represent diverse *V*_*H*_ to *DJ*_*H*_ joints. The same colours represent the same *DJ*_*H*_ joint and identical V*DJ*_*H*_ joints are not represented. Left lower panel: clone Xp40 flow cytometric profile as in **b**. Right lower panel: map showing polymorphisms in the *J*_*H*_ cluster of *Igh*^*a*^ (Balb/c) and *Igh*^*b*^ (C57BL/6) allotypes, the *J*_*H*_ segment position and relative positions of 3′-*J*_*H*_*1*, -*J*_*H*_*2* and -*J*_*H*_*3* primers (arrowheads, *J*_*H*_*4* primer position not shown in map).

Different scenarios could explain the distribution of the IgM^a^ and IgM^b^ percentages in CLP-derived clones (Fig. 3a). In the first scenario, the clones would be synchronized for V(D)J rearrangement and in each clone there would be a wave of IgM^a^ (or IgM^b^)-expressing cells followed by a partially overlapping second wave of IgM^b^ (or IgM^a^) cells. In this scenario, consistent with an asymmetric allele choice for rearrangement within each clone, the time point at which the clones were analysed would only be a snapshot, failing to capture the ongoing dynamics in the cultures. We have sampled the cultures at different time points and found no clear evidence for the existence of such waves in the majority of clones (Supplementary Fig. 5). The remaining scenarios can be distinguished by the status of rearrangement of the silent allele in the IgM^a^ and IgM^b^ populations of each clone. This status was estimated with the real-time quantitative PCR (qPCR) of the *V*_*H*_*–D*_*H*_ intergenic fragment and also by sequencing *V*_*H*_*DJ*_*H*_ and *DJ*_*H*_ rearrangements from the sorted populations, taking into account polymorphisms downstream of *J*_*H*_*1, J*_*H*_*2 and J*_*H*_*3* segments that enable us to assign rearrangements to the BALB/c (*Igh*^*a*^) or C57BL/6 (*Igh*^*b*^) allotypes. We first examined the CLP-derived clones with both the IgM^a^ and IgM^b^ pools clearly discernible and balanced with the help of flow cytometry (Fig. 4a,b). In the genome of these IgM^+^ populations, the percentage of *V*_*H*_*–D*_*H*_ intergenic fragment retention when compared with the Rag2^−/−^ genome was typically between 40% (similar to the mean retention observed in non-clonal *in vitro*-derived IgM^+^ cells, Fig. 4a) and 50% (the percentage in the genome of non-clonal *in vitro*-derived IgM^+^ cells of *Jht*^*+/−*^ mice). More importantly, within the same clone, the IgM^a^ and IgM^b^ genomes retain approximately the same percentage of the *V*_*H*_*–D*_*H*_ intergenic fragment, which means that each allele can undergo *V*_*H*_ to *DJ*_*H*_ rearrangement first (Figs 1 and 4a). Furthermore, we sequenced *DJ*_*H*_ and *V*_*H*_*DJ*_*H*_ rearrangements and found diverse *V*_*H*_–*DJ*_*H*_ junctions as well as diverse *D*_*H*_*–J*_*H*_ junctions, and a limited number of these *D*_*H*_*–J*_*H*_ junctions contributing to the diverse *V*_*H*_*DJ*_*H*_ rearrangements per clone. This shows that the Ly6d^+^ single-cell-sorted cells are devoid of *D*_*H*_*–J*_*H*_ rearrangements before culture, and suggests that in some clones we are testing subclonal expansions and tracking the choice to rearrange shortly before the decision to undergo *V*_*H*_ to *DJ*_*H*_ rearrangement. Representatively, in clone Xp40, the same two *DJ*_*H*_ junctions (one originating from the *Igh*^*b*^ allele and the other from the *Igh*^*a*^ allele) prevail in the genome of the IgM^a^, IgM^b^ and IgM^−^ pools (Table 2). The *Igh*^*b*^ *DJ*_*H*_ junction is shown to be predominantly contributing to the diverse *V*_*H*_*DJ*_*H*_^+^ rearrangements of the IgM^b+^ pool and in IgM^a^ pool the same *DJ*_*H*_ junction is part of non-productive (*V*_*H*_*DJ*_*H*_^−^) and the same junction was detected in the germline-*DJ*_*H*_ configuration, while the dominant *Igh*^*a*^ *DJ*_*H*_ junction was detected as part of productive (*V*_*H*_*DJ*_*H*_^+^) rearrangements in the IgM^a^ cell pool and in non-productive and *DJ*_*H*_ rearrangements in the IgM^b^ pool. Different *DJ*_*H*_ junctions are detected in all three fractions of the clone but were not detected as part of *V*_*H*_*DJ*_*H*_ rearrangements. From the analysis of rearrangements we conclude that the culture system mimics the features of *V*(D)J recombination *in vivo* and that considerable intraclonal diversity is generated in our clones, extending previous observations made in human B-cell lines^38^. More importantly, we observed that any of the alleles can undergo *V*_*H*_ to *DJ*_*H*_ rearrangement first within each clone.

### Founder effects in highly skewed clones

**Figure 5:**
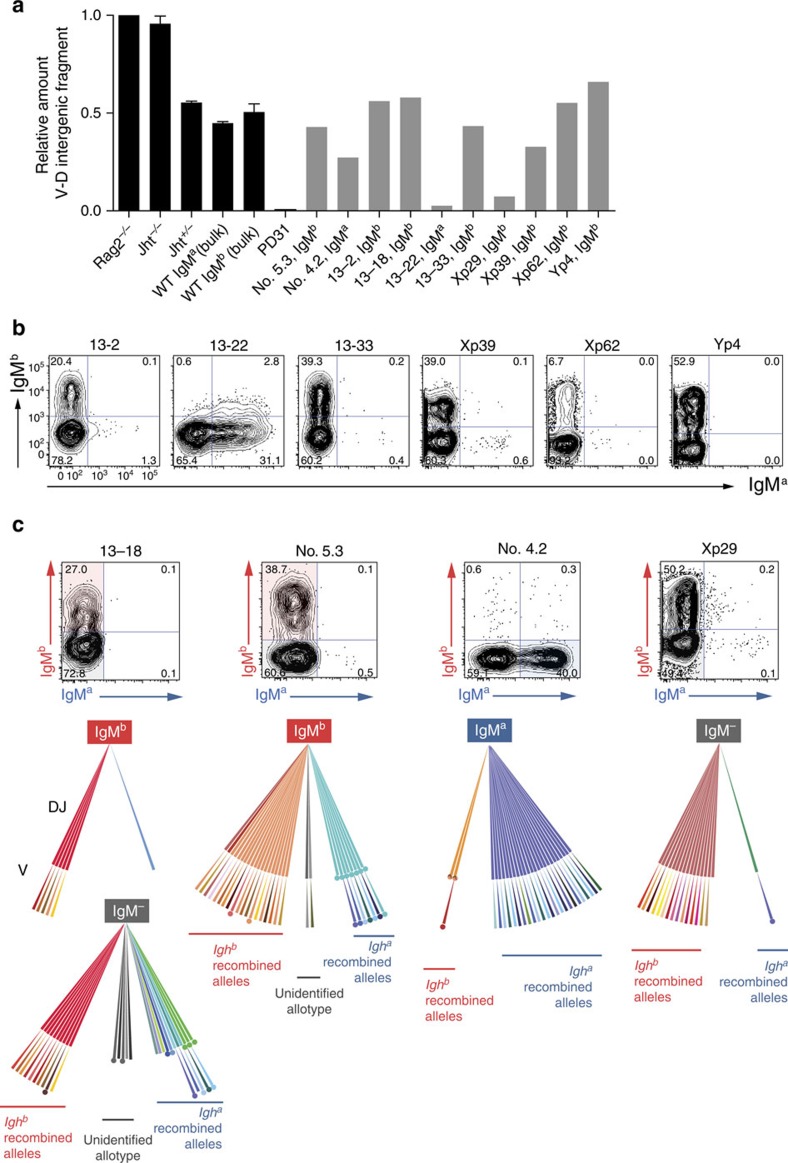
Rearrangement status of the silent *Igh* allele in highly skewed CLP-derived clones. (**a**) Quantitative PCR of the *V*_*H*_–D intergenic DNA fragment in IgM^+^ fractions of the *in vitro*-derived B-cell clones expressing only one allele (grey bars) and *in vitro*-derived controls. Error bars denote s.e.m. (**b**) CD19^+^-gated clones’ dot plots showing IgM^a^/IgM^b^ flow cytometric profiles for some of the clones in **a**. (**c**) Top panels’ dot plots show the remaining clones in **a**, not shown in **b**; shaded quadrants in the plots show the fractions that are represented in the tree-like scheme of the lower panels. Each scheme represents the diversity of *V*(D)J rearrangements obtained by sequencing V*DJ*_*H*_ and *DJ*_*H*_ rearrangements of the clone fractions indicated, as described in Fig. 4. From the represented clones, only clone 13–18 profile (for the IgM^+^ fraction) is compatible with a model of allelic preference. Clone no. 5.3 has a stop codon in the *Igh*^*a*^-derived *DJ*_*H*_ joint that prevents IgM^a^-productive rearrangements; clone no. 4.2 contains a prohibitive rearrangement in the *Igh*^*b*^-derived *DJ*_*H*_ joint (see Supplementary Table 6); clone Xp29 contains a single non-productive *V*_*H*_*DJ*_*H*_ rearrangement in the *Igh*^*a*^-derived allele as inferred by phenotype.

The most striking behaviour of the CLP-derived clones is the extreme bias displayed by some clones (Figs 3a,b and 5). This pattern could emerge from a pre-determined choice to rearrange a given allele first, if the culture conditions do not favour the second attempt to rearrange *V*_*H*_ to *DJ*_*H*_. The comparison between the amount of *V*_*H*_–*D*_*H*_ intergenic fragment in IgM^+^ non-clonal cell populations differentiated *in vivo* and *in vitro* shows that in culture conditions there is indeed a minor increase in the retention of the *V*_*H*_*–D*_*H*_ intergenic fragment (41.6±5.8% versus 34.1±4.9%), but a sizable proportion of cells have two rearrangements (Supplementary Fig. 5). Thus, it is difficult to explain the extreme skewing observed in these clones solely by attributing a fixed choice in the allele to undergo *V*_*H*_ to *DJ*_*H*_ rearrangement. Next, the status of rearrangement of the expressed and silent alleles in the clones was investigated. In the 18 IgM^+^ populations of the CLP-derived clones with both IgM^a^ and IgM^b^ clearly distinguishable (Fig. 4a), no example was found of a population with a *V*_*H*_–*D*_*H*_ intergenic fragment below 30%. However, in the 10 IgM^+^ populations from CLP-derived clones with extreme biases, three populations have a percentage of *V*_*H*_–*D*_*H*_ intergenic fragment below 30% (*P*=0.037, Fisher’s exact test). Interestingly, two of those clones were essentially depleted of the *V*_*H*_–*D*_*H*_ intergenic fragment (Fig. 5a), showing that these populations are mostly composed of cells that also have the silenced allele in the *V*_*H*_*DJ*_*H*_ configuration. This observation suggests that the extreme bias in this subset of clones results from a rearrangement occurring early on in the culture, that supposedly is non-productive and permanently silences the allele. In the case of the populations without a *V*_*H*_*–D*_*H*_ intergenic fragment, such rearrangement could be a *V*_*H*_*DJ*_*H*_^−^ or a *V*_*H*_*DJ*_*H*_^+^ encoding a μ chain that cannot form the pre-BCR^14^; in the case of the populations with a sizable *V*_*H*_–*D*_*H*_ intergenic fragment, such rearrangements could be *DJ*_*H*_ junctions using the reading frame 3 that frequently contains premature stop codons^39^. For the CLP-derived B-cell clone Xp29, which does not retain the *V*_*H*_–*D*_*H*_ intergenic fragment (Fig. 5a), sequencing data support the hypothesis of a non-productive rearrangement-imposed founder effect: the *V*_*H*_*DJ*_*H*_ rearrangements detected are diverse for the productive *V*_*H*_ to *DJ*_*H*_ junctions, but limited concerning both the *D*_*H*_ to *J*_*H*_ junctions (*N*=2 detected), and the *V*_*H*_ to *DJ*_*H*_ non-productive junction (unique sequence detected, several times; Fig. 5c and Supplementary Table 3). Thus, this clone appears to have made a first or very few non-productive *V*_*H*_ to *DJ*_*H*_ rearrangements early on in the culture, by chance in the same allele, which was followed by a number of cell divisions and then an extensive rearrangement of the second allele. Clones 13–33, Xp39, no. 5.3 and no. 4.2, which also show absolute biases (Fig. 5b) retain an intermediate amount of *V*_*H*_–*D*_*H*_ intergenic fragment (Fig. 5a). Sequencing data for clone no. 5.3 (showing a stop codon in the *DJ*_*H*_ joint of one of the alleles) and no. 4.2 (with a *DJ*_*H*_ joint that lacks a highly conserved codon) also confirm the founder effects imposed by rearrangement (Fig. 5c and Supplementary Tables 4 and 5). In clones 13-2, 13–18 Yp62 and Yp4, the silent allele is mostly in the *DJ*_*H*_ configuration (Fig. 5a), which suggests that one of the alleles may have a lower probability to rearrange. Sequencing analysis of the IgM^b^-skewed clone 13–18 indeed shows that the IgM^+^ population is composed of cells that have rearranged *V_H_* to *DJ_H_* only in the *Igh*^*b*^ allele, leaving *Igh*^*a*^ in the *DJ_H_* configuration (Fig. 5c and Supplementary Table 6). Interestingly, the IgM^−^ population sequencing analysis shows *V*_*H*_*DJ*_*H*_ rearrangements in both alleles, with *Igh*^*a*^ rearrangements that are more frequently non-productive (seven non-productive with frequent stop codons out of eight sequences) than the *Igh*^*b*^ ones (one non-productive out of 10 sequences). These results do not exclude that, for a few subclonal expansions, one of the alleles acquires an increased probability of rearrangement. Overall, we conclude that a minority of the CLP-derived clones with extreme skewing may have a relative bias to rearrange predominantly one of the alleles and that the majority of CLP-derived clones with extreme biases is explained by founder effects imposed by rearrangement that introduce a genetic constraint in the ability to rearrange a given allele in the precursor cells from which the IgM^+^ cells emerge.

## Discussion

Here we provide the first conclusive evidence that the allelic exclusion of immunoglobulin receptor loci differs from X-chromosome inactivation, as no stable epigenetic mark is propagated until pro-B cells start rearranging. More importantly, we also show that the *Igh* alleles rearrange independently from each other in the majority of clones derived from common lymphoid precursors.

Roughly, the number of cells expressing the maternal or the paternal *Igh* allele is the same. Similarly, in female mammals each cell inactivates the paternal or the maternal X-chromosome with identical probability. This common feature is frequently emphasized, and it has been hypothesized that autosomal genes under monoallelic expression of one or the other allele are flanked by regions with a high density of long interspersed nuclear elements^40^, the same elements that play a key role in the generation and propagation of the silencing epigenetic marks along the X-chromosome^41^. Furthermore, it is known that the alleles replicate asynchronously in parent-of-origin imprinting^42^, other cases of monoallelic expression^28^,^43^ and X-linked inactivation^44^. In particular, antigen receptor loci were found to establish this pattern early in development, around the time X-inactivation is also established, and then proceed to maintain it^28^. This led to the proposal that the allele to replicate first retains its epigenetic mark as the cell divides and undergoes differentiation, so that in clonally related developing B cells, it is always the same allele that undergoes *V*_*H*_ to *DJ*_*H*_ rearrangement first^28^. The analysis of haematopoietic systems resulting from a single female HSC is a direct test of this hypothesis because the HSC population is heterogeneous for the inactivated X-chromosome, and if the *Igh* locus carries an epigenetic mark that is transmitted to the progeny as stably as the X-chromosome marks, *V*_*H*_ to *DJ*_*H*_ rearrangement would occur first in the same allele in all, or the vast majority, of the pro-B cells. As expected^45^, X-chromosome inactivation is stably transmitted during all cell divisions that occur as a HSC differentiates into a regulatory T cell; in contrast, from the status of rearrangement of the silent allele in IgM^a^- and IgM^b^-sorted populations we have found that in cells clonally related to a single HSC, either the maternal or the paternal alleles can rearrange the *V*_*H*_ first with similar frequency, thus showing that there is no stable epigenetic mark established early in development that defines a temporal allelic hierarchy for *V*_*H*_ to *DJ*_*H*_ rearrangement. Farago *et al.*^46^ have reported the same conclusion for the *Igκ* locus. In their study, multiple HSCs carrying distinguishable *Igκ* alleles (hC*κ*/mC*κ*) were transduced with lentiviruses (that integrate the genome of each cell in a unique manner, thus identifying its clonal progeny), and then used in bulk to reconstitute mice. This technique was instrumental in the past to establish whether cell lineages share a precursor cell^47^; however, we argue that it provides inconclusive results for allelic commitment. Since it is well established that 40–50% of B cells rearrange both *Igκ* alleles^48^, the progeny of each precursor cell is inevitably present in both the hCκ^+^ and mCκ^+^ populations and it follows that the pattern of lentiviral integrations in the isolated hCκ^+^ and mCκ^+^ populations is expected to be roughly the same, with or without commitment. Instead, from the *in vivo* clonal analysis here described based on the analysis of the status of rearrangement of the silent allele, we conclude that the parallel between the allelic exclusion of *Igh* and X-chromosome inactivation is misleading. It thus appears that either allele from the daughter pro-B cells of a given HSC can undergo *V*_*H*_ to *DJ*_*H*_ first, and that asynchronous replication is not a good predictor of the fate of the alleles, in *V*(D)J recombination and other contexts. Indeed, it has been shown that replication timing is stable after X-inactivation but switches between alleles before the onset of the inactivation and is independent from the subsequent choice to inactivate the X-chromosome^49^.

The data from CLP-derived clones revealed that both *Igh* alleles have the potential to undergo *V*_*H*_ to *DJ*_*H*_ first, until shortly before the onset of rearrangement. It cannot be completely excluded that the accessibility of the Igh locus in culture may differ from the *in vivo* conditions, particularly at the level of the initiation of the *Igh* locus accessibility, nuclear localization of the alleles and overall rearrangement kinetics. However, our culture conditions are consensually accepted as mimicking the B-cell development that occurs *in vivo* in the bone marrow^34^, allelic exclusion was observed in the flow cytometric profiles of the clones, *in vitro* experiments performed months apart in different laboratories with cytokine supernatants from different batches produced similar results and a direct comparison of rearrangement efficiencies *in vitro* and *in vivo* did not reveal statistically significant differences (Supplementary Fig. 6). Strikingly, most of the clones with an extreme bias in the expression of IgM^a^ or IgM^b^ could be explained as a consequence of rearrangement dead ends, that is, those that permanently prevent one allele to be productively expressed at the membrane, giving the other the chance to undergo multiple productive rearrangements within the clone. This explanation of a bias determined by a limiting number of rearranging cells is compatible with the more balanced IgM^a^/IgM^b^ ratios in clones derived from HSCs and MPPs, in which rearrangement starts relatively late in the cultures, after the initial cell has expanded substantially. It also explains the slightly more pronounced IgM^a^/IgM^b^ bias in Ly6d^+^-derived clones compared with the clones derived from the precursor Ly6d^−^, since the former start rearranging in culture earlier than the latter. Finally, it makes sense that, in CLP-derived clones, the allele rearrangement bias observed for the *Igh* alleles is not reproduced by the *Igκ* alleles, which typically rearrange after the substantial expansion of cells expressing the pre-BCR^37^,^50^. This last result is the only one addressing the *Igκ* locus in this work and does not reproduce the results presented in ref. 46. Further analysis is needed to address this issue thoroughly.

From these results, it can be concluded that the mechanism of allelic exclusion of the *Igh* locus differs from the one claimed for the *Igκ* locus^46^. In that study, it was proposed that stable epigenetic marks acquired around the time of lymphoid differentiation pre-determine a temporal hierarchy for rearrangement; however, the status of rearrangement of the silenced allele in the clones analysed was not reported, which limits the interpretation of the data. It is nevertheless possible that the *Igh* and the *Igκ* loci behave differently, particularly given the organization of the *V*_*H*_ and *J*_*H*_ elements that allows successive rearrangements in the *Igκ* alleles and the existence of *D*_*H*_ elements in the *Igh* locus. Indeed, the *Igh* locus has more structural similarities with the *Tcrβ* locus than with the *Igκ* locus, namely the ordered rearrangement. Of note, the *Tcrβ* alleles were found to independently associate with pericentromeric heterochromatin^51^, an interaction that inhibits rearrangement, and this observation is compatible with our conclusions for the *Igh* locus. Furthermore, this model is also the simplest explanation. Unlike the ‘pre-determination’ model, in which the strict temporal hierarchy for recombination implies an *ad hoc* mechanism to render the second allele active for recombination, in the model of independent recruitment the second rearrangement will eventually occur unless the first rearrangement triggers the feedback mechanism that shuts down recombination or the cell dies. The idea of independent recruitment is also compatible with the existence of a proportion of cells with ‘synchronous’ rearrangements, that is, cells in which the second *V*_*H*_ to *DJ*_*H*_ rearrangement occurs in the time window before the feedback mechanism triggered by the formation of the pre-BCR blocks further rearrangement of the *Igh* locus (Supplementary Fig. 7). It is not known what proportion of cells rearrange the alleles ‘synchronously’. However, in the absence of complementary feedback mechanisms mediated by ATM^3^ and the μ chain messenger RNA^17^, which respond to the first rearrangement faster than the feedback triggered by the pre-BCR, there is a measurable, although small, increase in the frequency of allelically included cells. We have confirmed this increase in genomic allelic inclusion for *Atm*^*−/−*^ cells in our assay (Fig. 2c). Finally, this model of independent rearrangement is not incompatible with the existence of moderate and transient allelic biases in the probability to undergo *V*_*H*_ to *DJ*_*H*_ rearrangement, if each allele behaves independently from the other while becoming accessible to rearrangement, but not from its previous copy in the mother cell. Thus, the simplest interpretation of our data is that in the vast majority of clones the two alleles gain recombination competence in a sufficiently synchronized way for the V(D)J mechanism to reveal its stochastic component. Any case of an initial asymmetry that may persist for a number of divisions and lead to biases in the recombination does not contradict the model. Indeed, we may have identified such possible bias in only one of the clones studied, and these rare cases are expected from a truly independent opening of the alleles (Supplementary Fig. 7).

For over three decades, the explanations of allelic exclusion have essentially differed on the weight given to the stochastic and the regulated events. Using *in vivo* and *in vitro* clonal approaches that are based on the outcome of the rearrangement process in genetically unmanipulated murine cells, we found that an independent recruitment of the *Igh* alleles underlies the mechanism of allelic exclusion of the *Igh* locus. This intrinsic stochastic feature establishes the default mechanism that ensures two rearrangement attempts per cell, unless the first rearrangement triggers the definitive feedback that shuts down further rearrangement.

## Methods

### Mice

All mice were bred and maintained at the specific pathogen-free animal facilities of the *Instituto Gulbenkian de Ciência* (Oeiras, Portugal). BALB/c, C57BL/6J, B6.Rag2^−/−^, B6.SJL-Ptprc^a^ (the pan-leukocytic marker gene *Ptprc*^*a*^ is also known as *CD45.1* or *Ly5.1*; the C57BL/6J strain carries the *Ptprc*^*b*^ allele, also known as *CD45.2* or *Ly*^*5.2*^) and B6.Cg-Igh^a^ were originally received from The Jackson Laboratory (Bar Harbor, ME, USA). Mice with *Foxp3*^*gfp*^ knock-in^31^ (B6.*Foxp3*^*gfp*^, courtesy of Dr Alexander Rudensky—Memorial Sloan-Kettering Cancer Center, New York, USA) were backcrossed with the C57BL/6J strain at our animal facility. Animals were bred to generate female heterozygous B6.Cg-CD45.1/45.2*Igh*^*a/b*^*Foxp3*^*gfp/wt*^ and used in cell transfer experiments. Female 8–12 week-old-mice were used as donors. For the *in vitro* cell culture system (BALB/c x C57BL/6J)F1, B6.Cg-*Igh*^*a/b*^ or B6;129.*Igh*^*a/b*^*Igk*^*h/m*^ mice heterozygous for the *Igh* and/or the *Igκ* loci were used (B6;129.*Igκ*^*h*^ mice^52^ were courtesy of Dr Michel Nussenzweig (Rockefeller University, NY, USA). Mice with a targeted deletion for the immunoglobulin heavy chain joining region (B6.Igh-Jht^−/−^ mice^33^) were courtesy of Dr Jocelyne Demengeot—IGC, Oeiras, Portugal) or ATM knockout mice (B6;129.*Atm*^−/−^ mice^53^, courtesy of Dr Almudena Ramiro—CNIO, Madrid, Spain). This research project was reviewed and approved by the Ethics Committee of the Instituto Gulbenkian de Ciência, and by the Portuguese National Entity that regulates the use of laboratory animals (Direcção Geral de Alimentação e Veterinária. All experiments conducted on animals followed the Portuguese (Decreto-Lei n° 113/2013) and European (Directive 2010/63/EU) legislations concerning housing, husbandry and animal welfare.

### Cell suspensions

All cells were suspended in Hanks’ balanced-salt solution (HBSS, Gibco) supplemented with 5% fetal bovine serum (FBS, Life Technologies). BMs were flushed and single-cell-suspended from the femora and tibiae with a 26-gauge needle of 1-ml syringe, filtered and rinsed with HBSS 5% FBS. Thymi, spleens and lymph nodes were dissected and single-cell suspensions obtained using a 70-μm nylon mesh. Blood was collected from the facial vein in EDTA. Erythrocytes from blood and spleen samples were lysed using an NH_4_Cl hypotonic solution at a 9:1 NH_4_Cl:cell suspension ratio (no. 07850, STEMCELL Technologies), for 10 min and immediately rinsed at least twice and resuspended in HBSS with 5% FBS.

### SP staining

BM cell suspensions were incubated with the FcBlock reagent (anti-CD16/32, in Rat serum—no. 13551, STEMCELL Technologies), labelled with the biotinylated antibodies anti-CD45R/B220, anti-CD19, anti-CD11b/Mac-1, anti-Ly6G/GR-1, anti-pan-NK, anti-Ly-76/TER and anti-CD3 (full list of antibodies and dyes in Supplementary Table 1), and incubated with Streptavidin microbeads (Miltenyi Biotec), for negative selection of lineage-positive cells by immunomagnetic separation using a MACS column (Miltenyi Biotec). Cells were further incubated in serum-free media (StemSpam SFEM, no. 09650, StemCell Technologies) with 20 μg ml^−1^ Rhodamine 123 (no. R302, Invitrogen) for 30 min in a water bath at 37 °C (in the dark), cooled, rinsed with HBSS, 5% FBS and then stained with Hoechst 33342 (no. H3570, Invitrogen) at a concentration of 2.5 μg ml^−1^ diluted in the serum-free media as before, for 90 min at 37 °C, in the dark. Cells were then labelled in HBSS with 5% FBS for 30 min on ice, with Streptavidin-APC and anti-CD45-PE and 1 μg ml^−1^ Propidium Iodide (no. P3566, Invitrogen). For the long-term reconstituting SP cells gating adjustment, an aliquot of the sample was incubated with 25 mg ml^−1^ of Verapamil (no. 676777, Calbiochem) before staining with the dyes. Further stainings with different HSC-specific marker combinations were tested, confirming the enrichment of HSCs within the SP gates.

### Cell sorting and *in vivo* single-cell repopulation assay

Single Lin^−^Rho^−^CD45^int^Hoe^−^ long-term reconstituting SP cells of adult female mice in the C57BL/6J background, heterozygous for *CD45.1/45.2*, *Foxp3*^*gfp/x*^ and *Igh*^*a/b*^, stained as above described, were sorted using the single-cell deposition unit of a MoFlo (Beckman Coulter) or FACSAria (Becton Dickinson) fluorescence-activated cell sorter machine into the individual wells of Terasaki plates (no. 452256, MicroWell 60-well MiniTray, Nunc Brand, Thermo Fisher Scientific Inc.) preloaded with 15 μl of serum-free media (StemSpam SFEM, StemCell Technologies). Sorted IgM^a^ and IgM^b^ populations had purities above 95% (for most samples the purity was assessed with flow cytometry immediately after the sorting and for a few samples the purity was assessed with flow cytometry and/or semiquantitative PCR analysis of an allele-specific polymorphism in the *VH*–D intergenic region). To check for the purity of the single-cell sorting used in the *in vivo* experiments, each well was examined in a 4 °C room using an inverted microscope and the wells containing single cells were used in reconstitutions (Supplementary Fig. 1).

Eight- to twelve-week-old recipient females of the B6.Rag2^−/−^ background received sublethal whole-body γ-irradiation with 600 cGy (Gammacell 2000 Mølsgaard Medical), 4–8 h before an intravenous retro-orbital injection with one or more test cells, as indicated. Recipient animals were analysed routinely every 2 weeks for up to 10 weeks post reconstitution for the presence of chimeric cells in the peripheral blood by staining with anti-CD45.1 and anti-CD45.2 antibodies (Supplementary Table 1). Clonally repopulated animals were killed and cells from BM, spleen, thymus and lymph nodes (LNs) were analysed for the presence of terminally differentiated B cells, T cells and myeloid cells of donor origin, as well as progenitor donor cells (antibodies used are described in Supplementary Table 1). Animals selected for subsequent analysis showed in Tregs *Foxp3*^*gfp*^ knock-in gene expression from a single X-chromosome. Splenic CD19^+^IgM^a+^ and IgM^b+^ cells were then sorted on a FACSAria instrument, their DNA extracted and the recombination status of the *Igh* gene was analysed as described below.

All flow cytometric data collection was performed on a FACSCalibur (Becton Dickinson), a FACSAria (Becton Dickinson) or a CyAN ADP (Dako Cytomation, Beckman Coulter). The analysis was performed using the FlowJo software (Tree Star Inc.).

### *In vitro* B-cell clonal differentiation from precursors

BM cell suspensions enriched for haematopoietic progenitors (Lin^−^) were treated as described in ref. 34. Briefly, HSC, MPP and CLP cells were sorted using a FACSAria instrument after staining with anti-CD117(c-Kit), anti-Ly6A/E(Sca-1), anti-CD127(IL7Ralpha) and anti-CD135(FLK2; Supplementary Table 1). Single cells were directly sorted into flat-bottom 96-well plates (Techno Plastic Products) previously seeded with non-irradiated 2.5 × 10^3^ OP-9 cells per well, in complete culture media (OPTI-MEM, Invitrogen), supplemented with 10% FBS (Life Technologies) penicillin (50 U ml^−1^), streptomycin (50 μg ml^−1^), β-mercaptoethanol (50 μM) and Gentamycin (50 μg ml^−1^) and cultured in saturating amounts of the following cytokines: interleukin-7 (IL-7), c-Kit ligand and Flt3 ligand. Supernatants of myeloma cell lines transfected with the DNA encoding the above-mentioned cytokines were kindly provided by Dr Ana Cumano, Institut Pasteur, Paris, France.

After 12–14, 17–20 or 34 days of culture of CLPs, MPPs or HSCs, respectively, individual plates were analysed for single colonies. Wells with single colonies were stained with anti-CD19, anti-IgM^a^ and anti-IgM^b^ (Supplementary Table 1) analysed with flow cytometry and each IgM^+^ allelic fraction (and in some cases also the IgM^−^ fraction) was sorted in bulk using a FACSAria cell sorter instrument as described above.

### DNA samples

Genomic DNA (gDNA) from *in vivo*-differentiated clonal CD19^+^IgM^a^ and CD19^+^IgM^b^ pools of at least 10^4^ sorted cells was isolated by proteinase K (Sigma) digestion, phenol–chloroform-extracted, precipitated by ethanol and resuspended in TE buffer. The set of *in vitro*-differentiated sorted samples included also CD19^+^IgM^−^ populations and the numbers of sorted cells were invariably smaller than those of the *ex vivo* samples (at least 2,000 cells); for these *in vitro*-derived samples, gDNA was obtained by incubation with cell digestion medium containing 400 ng μl^−1^ proteinase K and 17 μM sodium dodecyl sulfate (SDS, Sigma) in PBS (Invitrogen) for 1 h at 50 °C, followed by enzyme heat inactivation for 30 min at 95 °C. gDNA thus obtained was diluted to homogeneous cell-equivalents per μl, as determined by the number of cells sorted, and used directly in PCR reactions, as described below. Treatment of control samples was matched to that of each set of samples. As controls for *in vivo*-differentiated clones, *ex vivo* splenocytes of (C57BL/6*BALB/c)F1 WT animals and mice of the *Rag2*^−/−^, *Jht*^−/−^, *Jht*^+/−^ and *Atm*^−/−^ genotype were sorted and treated as the test samples. qDNA from the cells lines 18–81 and PD31 (cell lines kindly provided by Dr Michele Goodhardt, Institut Universitaire d’Hématologie, Paris, France and Dr Patricia Cortes, Icahn Medical Institute, New York, USA) were used as negative controls for the presence of *V*_*H*_–*D*_*H*_ intergenic fragment.

### Real-time quantitative PCR

The frequency of *DJ*_*H*_ only rearranged alleles in the IgM^a^ and IgM^b^ populations of the same B-cell clones was determined with real-time qPCR, with the test and reference sequences localized in the recombining and non-recombining regions of the *Igh* locus, as shown in Supplementary Fig. 2a. The test primer pairs, VDi4FW_5′-GCAGGAAGACCACAGGAGC-3′ and VDi4RE_5′-AGCCACTGTAGATGAGGGGT-3′, amplify a ~100-bp fragment herein termed ‘*V_H_*–D intergenic fragment’ downstream of the last *V_H_*-gene segment of the *Igh* locus and upstream of the first D_H_-gene segment. The reference primer pairs, CmuFW_5′-GTTCTGTGCCTCCGTCTAGC-3′ and CmuRE_5′-CTTGGGGTGACTGAGCATTTGC-3′, also amplify a ~100-bp fragment located in the μ-constant region of the *Igh* locus (*Cμ*). Each primer pair was included in a different reaction mixture and runs were performed in triplicates or quadruplicates using the 7900HT Fast Real-Time PCR System (Applied Biosystems), in 384-well plates with optical tape sealers (Applied Biosystems). Reactions were performed in 10 μl final volume containing 1 × the Power SYBR Green PCR Master Mix (Applied Biosystems) and 0.2 μM of each primer. Thermocycling conditions were 95 °C for 10 min, followed by 45 cycles of 95 °C for 15 s and 60 °C for 60 s. The melting curve of the finished PCR reaction was systematically used to confirm that a single amplicon was amplified.

Calibration curves were performed using six standard dilutions, in triplicates, of B6.Jht^+/−^ gDNA from *ex vivo*-sorted IgM^+^ cells, ranging from 17 cell equivalents up to 54,240 cell equivalents per reaction (Supplementary Fig. 2b), and test sample concentrations used per run ranged from 200 to 700 cell equivalents per run. No template control reactions were performed using water or a diluted digestion medium solution without template. Beyond replicas, independent repetition of each sample quantification was performed whenever the sample amount was sufficient, in diverse dilutions, but given the low amount of DNA in the majority of samples it was not possible to repeat the PCR on a different day. The SDS software v.2.2 (Applied Biosystems) was used to calculate the quantification cycle (*C*q) value. Real-time quantification data were averaged between replicates and relative amount of DNA was calculated with the *Cμ* constant fragment quantification as the reference, using the delta *C*q method adjusted for PCR efficiency^54^.

### *Igh* rearrangement PCR and sequencing

For the amplification of *DJ*_*H*_ and *V*_*H*_*DJ*_*H*_ rearrangements, we used the primer collection and the two-step nested PCR conditions previously described^18^. One thousand cell-equivalents per run were used for the first round multiplex PCR of *V*_*H*_*DJ*_*H*_-rearrangement amplifications in a 100-μl final master mix volume. For the nested round of PCR, alternative reverse primers were *J*_*H*_*1-*, *J*_*H*_*2-* and *J*_*H*_*3*-specific (JH1A—5′-TGTGGCAGATGGCCTGACATGGGG-3′, JH2A-5′-TCAGCCAGGGCTCCCAATGACCC-3′, and JH3A-5′-CAGCAGGCAGAGAGTCCCTGACCC-3′), allowing allotype discrimination upon sequencing (Supplementary Fig. 3). For sequencing, PCR bands were cloned in the pGEM-T Easy VECTOR SYSTEM (Promega) and a number of clones were analysed. Typically, four clones were sequenced per band. Given both the high number of PCR cycles used and the cloning step before sequencing the *V*_*H*_*DJ*_*H*_ amplicons, it was necessary to evaluate the impact in the analysis of PCR/sequencing-introduced errors. This was estimated using the formula (*f*_mut_ × *p*_stop_+*f*_indel_−*f*_mut_ × *f*_stop_ × *f*_indel_) × *L*/1,000, where *f*_mut_ and *f*_indel_ stand for the number of PCR/sequencing-introduced point mutations (2.6) and the number of PCR/sequencing-introduced gaps/insertions (0.18) per 1,000 nucleotides, respectively, estimated from alignments of the germline regions (a total of 16,020 bp) downstream of the *J*_*H*_ elements that we have sequenced, *p*_stop_ is the probability of producing a stop codon from a PCR-introduced point mutation (0.071) taking in consideration the codon usage of the *V*_*H*_*DJ*_*H*_ sequences obtained in this study and *L* is the average size of the amplicons. If the entire *V*_*H*_ and CDR3 regions (*L*=269 bp) of the amplicon are considered, the percentage of productive *V*_*H*_*DJ*_*H*_ rearrangements that become non-productive because of PCR errors is 8.9%. This percentage decreases to 2.0% if only a sequence region including the entire CDR3 is used (*L*=60 bp), which is feasible since the PCR errors in the *V*_*H*_ region can be aligned with the known *V*_*H*_ germline sequences from antigen receptor databases. In any case, the errors introduced by PCR do not affect our conclusions.

## Author contributions

C.F.A.P. and V.M.B. designed the experiments, analysed the data, discussed the results and wrote the manuscript. C.F.A.P. performed all the experiments. R.de.F and F.M. provided technical assistance. T.L. and R.G. gave support concerning flow cytometry. P.V. advised and gave support to all experiments. V.M.B. supervised all the work.

## Additional information

**How to cite this article**: Pereira, C. F. A. *et al.* Independent recruitment of *Igh* alleles in V(D)J recombination. *Nat. Commun.* 5:5623 doi: 10.1038/ncomms6623 (2014).

## Supplementary Material

Supplementary Information Supplementary Figures 1-7, Supplementary Tables 1-6 and Supplementary References.

## Figures and Tables

**Table 1:** Single HSC-reconstituted animals and representative controls.

	**Sample (no.)**	**No. injected cells**	**Graft age (weeks)**	**(%) CD45.1**^+^ **spleen**	**(%) CD45.1**^+^ **LN**	**(%) CD45.1**^+^ **BM**	**(%) CD45.1**^+^ **thymus**	**FOXP3**^−^ **GFP**^−^	**FOXP3**^−^ **GFP**^+^
**1.**	4.1_2.7M	1	10.8	26.6	14.6	1.6	2.9	0.0	100.0
**2.**	4.2_2.7M	1	10.8	18.6	32.5	2.4	1.8	0.0	100.0
**3.**	5.1_3.5M	1	14.0	71.0	27.8	73.6	—	0.6	99.4
**4.**	5.2_3.5M	1	14.0	24.9	89.9	10.9	46.4	99.6	0.4
**5.**	8.1_3.7M	1	14.8	59.9	69.5	2.3	0.1	98.3	1.7
**6.**	4.6_4.0M	1	16.0	41.6	12.4	2.3	3.0	0.0	100.0
**7.**	4.3_4.5M	1	18.0	15.4	1.6	1.3	0.1	—	—
**8.**	4.5_4.5M	1	18.0	23.9	72.3	1.6	3.7	100.0	0.0
**9.**	8.2_5.4M	1	21.6	39.3	3.1	17.8	2.6	0.0	100.0
**10.**	8.3_5.4M	1	21.6	35.7	—	3.2	6.3	—	—
**11.**	8.4_5.4M	1	21.6	32.7	55.1	4.1	32.6	0.6	99.4
**12.**	8.5_5.4M	1	21.6	—	59.8	6.2	18.8	99.6	0.4
**13.**	8.6_5.4M	1	21.6	19.4	—	7.2	7.6	—	—
**14.**	8.7_5.4M	1	21.6	37.5	—	6.4	19.2	—	—
**15.**	5.4_6.9M	1	27.4	76.7	82.1	13.6	8.6	99.8	0.2
**16.**	4.1_7.9M_2nd_a	1	31.6	1.6	2.1	0.6	30.8	0.0	100.0
**17.**	4.1_7.9M_2nd_b	1	31.6	8.1	50.7	1.4	1.4	0.6	99.4
**18.**	5.1_7.4M_2nd_a	1	29.6	37.1	68.1	2.6	6.1	0.8	99.2
**19.**	c4.7_2.2M	6	8.8	—	73.1	—	12.5	99.5	0.5
**20.**	c4.4_4.5M	6	18.0	51.5	25.0	13.0	0.2	100.0	0.0
**21.**	c4.12_7.9M	12	31.6	18.2	22.3	0.3	0.3	—	—
**22.**	c8.49_3.7M	<50	14.8	78.9	77.6	14.3	72.8	97.4	2.6
**22.**	c4.6_2.2M	50	8.8	27.0	78.9	—	57.3	6.5	93.5
**23.**	c8.50_3.7M	50	14.8	25.7	82.9	12.1	0.2	13.4	86.6
**24.**	c5.1000_2.7M	1,000	10.8	69.7	19.4	13.9	69.6	42.4	57.6
**25.**	B6.Foxp3^wt/wt^	GFP^−/−^ Control	—	—	—	—	—	99.9	0.1
**26.**	B6.Foxp3^gfp/gfp^	GFP^+/+^ Control	—	—	—	—	—	0.2	99.8
**27.**	B6.Foxp3^gfp/wt^	Donor Control	—	—	—	—	—	53.5	46.5

BM, bone marrow; CWBM, clonal whole bone marrow; GFP, green fluorescent protein; HSC, haematopoietic stem cell; LN, lymph node.

Entries 1–15 correspond to single-cell-injected mice, 16–18 are secondary recipients of 5,000 WBM cells of clone no. 4.1_2.7 M (line 1) and no. 5.1_3.5 M (line 3), lines 19–24 correspond to animals injected with more than one cell, and animals 25–27 are non-reconstituted controls. Graft age is the time in weeks at which the sample was collected after the injection of the single cell. Frequency of CD45.1^+^ cells in the spleen, LN, BM and thymus is presented if available. The last two columns (Foxp3−GFP^+/−^) show the frequency of LN CD45.1^+^Foxp3^+^ cells that were staining positive or negative for Foxp3−GFP+ by flow cytometry (representative histograms are shown in Fig. 2a). The animal no. 8.1_3.7 M was excluded from further analysis because a small population of GFP+ cells were observed in addition to GFP−Foxp3+ cells. On the basis of these data, we estimate that the percentage of false monoclonal reconstitutions within the selected pool of animals with unimodal distributions of GFP in the regulatory T-cell population is below 5%.

**Table 2:** Sequencing *V*_*H*_*DJ*_*H*_ and *DJ*_*H*_ junctions of clone Xp40.
