# Supplementary material for: Independent recruitment of Igh alleles in V(D)J recombination
Source: Nat Commun. 2014 Dec 17;5:5623. doi: 10.1038/ncomms6623 (PMC4351640; doi:10.1038/ncomms6623)
Supplement: Supplementary Information — Supplementary Figures 1-7, Supplementary Tables 1-6 and Supplementary References. [file ncomms6623-s1.pdf]

Supplementary Information

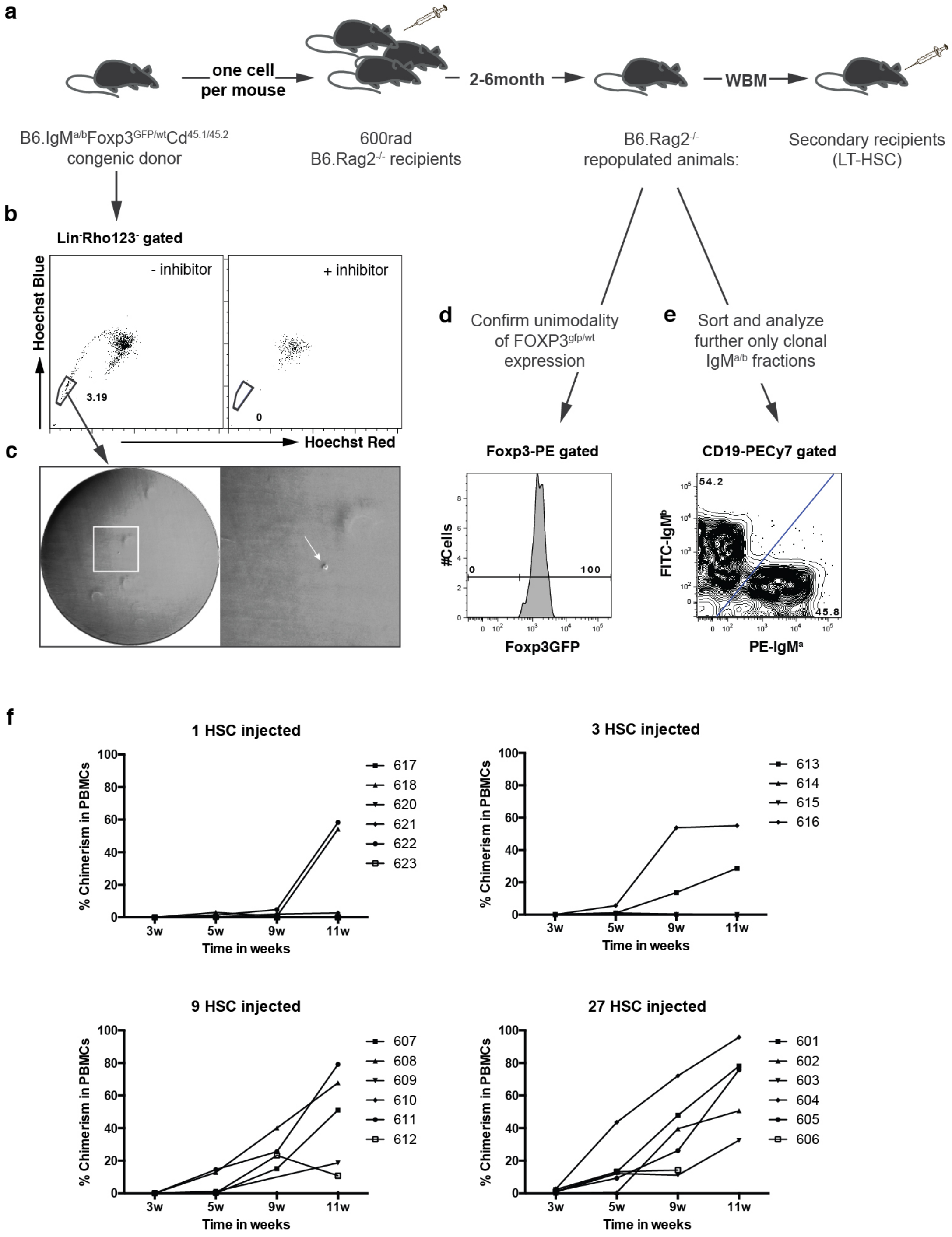

Supplementary Figure 1. Experimental set-up (related to Methods and Table 1). (a) Single cell injection scheme for clonally repopulating mice; donor mice were of the C57BL/6J background and heterozygous for selected loci

($Cd^{45.1/45.2}Foxp3^{gfp/wt}Igh^{a/b}$); recipients of the B6-Rag2$^{-/-}$ background were sub-lethally gamma-irradiated (600 cGy) and injected with a single WT donor cell; BM from positively selected mice were tested for secondary repopulation capabilities by re-injection of a fraction of the whole bone marrow or in some cases lineage-depleted BM in irradiated B6-Rag2$^{-/-}$ mice. (b) Representative plots for the side-population (SP; positive and negative control – sample treated with verapamil inhibitor) and (c) photograph of a Terasaki well under an inverted microscope and a zoom-in detail of the single Lin^{-}Rho^{-}CD45^{int}Hoe^{-} SP cell sorted. (d) Injected animals were sacrificed (starting at 8 up to 18 weeks from injection) and the chimeric ones were screened for monoclonality based on the GFP expression in FOXP*3*$^{+}$ T cells from LN. (e) B-cells from spleens were sorted according to IgM^{a}/IgM^{b} expression and used for further analysis. (f) Titration of the repopulation potential. Chimerism frequency was measured in peripheral blood mononuclear cells (PBMCs), calculated as the number of CD45.1/45.2 donor cells divided by the sum of the number of CD45.1/45.2 (donor) + CD45.2/45.2 (recipient) cells x100 detected on a FACSCalibur instrument after PBMCs staining with specific fluorescent-labeled antibodies (see Methods).

a

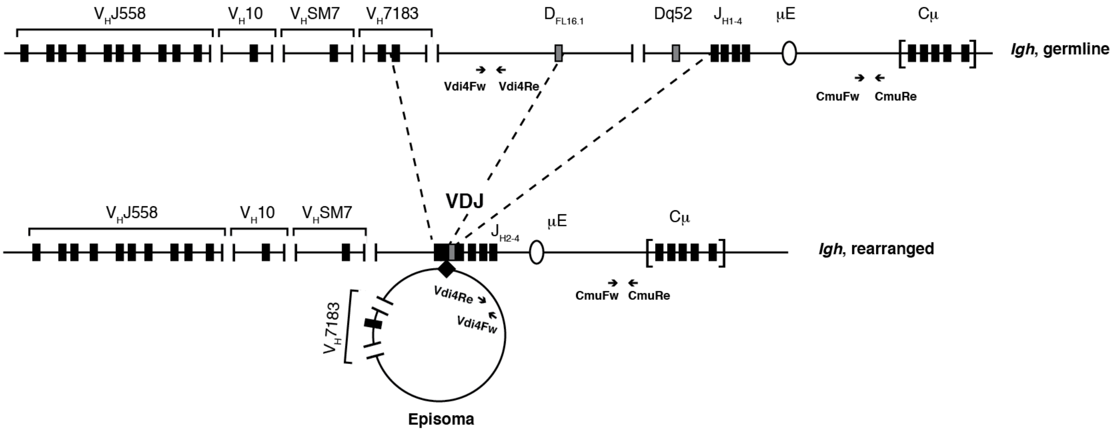

b

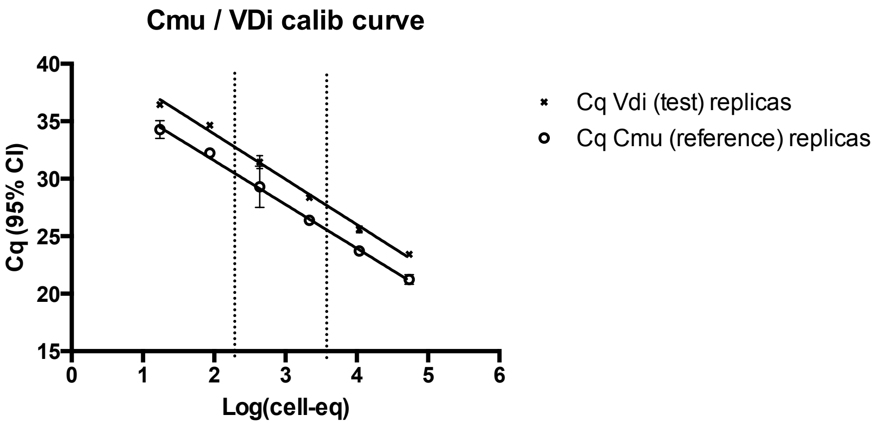

	Cq Vdi (test) replicates	Cq Cmu (reference) replicates
Best-fit values		
Slope	-3.938 ± 0.08725	-3.813 ± 0.04912
Y-intercept when X=0.0	41.77 ± 0.3062	39.20 ± 0.1656
X-intercept when Y=0.0	10.61	10.28
1/slope	-0.2539	-0.2622
95% Confidence Intervals		
Slope	-4.128 to -3.748	-3.921 to -3.705
Y-intercept when X=0.0	41.10 to 42.44	38.84 to 39.57
X-intercept when Y=0.0	10.26 to 10.99	10.08 to 10.49
Goodness of Fit		
r squared	0.9941	0.9982
PCR efficiency=	1.79	1.83

Supplementary Figure 2. Quantitative PCR (related to Methods). (a) Scheme of the *Igh* locus with the positions of qPCR primers pair for the V_H-D_H intergenic fragment quantification (Vdi4Fw/Vdi4Re) and reference pair (CmuFw/CmuR): only reference primer pairs amplify when V_H to DJ_H rearrangement has occurred and the episome has been diluted in the cells through cell proliferation. (b) Calibration

curves. Vertical lines mark the range of concentrations used in the analysis of the *in vivo* and *in vitro* samples.

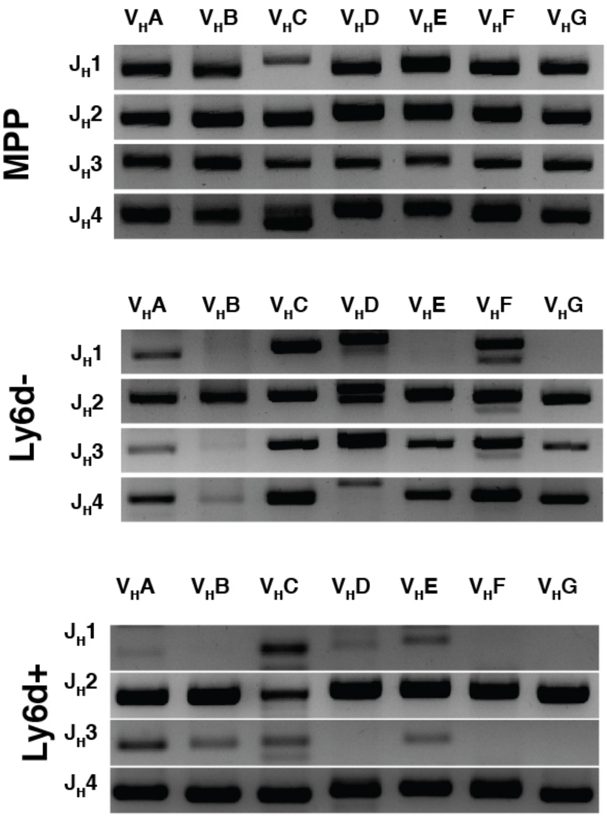

Supplementary Figure 3. Amplification of *Igh* rearrangements with V_H-specific degenerate primers and J_H sequence polymorphisms map (related to Methods). (*In vitro* intraclonal B cell rearrangement diversity is higher in the MPP-derived clones than in the $Ly6d^-$ and $Ly6d^+$-derived CLP clones, as shown by comparing the number of V(D)J rearranged amplicons in agarose gels (1.5%). Double negative fractions ($CD19^+IgM^-$ fraction) of MPP, CLP.$Ly6d^-$ and CLP.$Ly6d^+$ of *in vitro*-derived B-cell clones were PCR-amplified with 5' primers degenerated for V_H family genes (primers V_HA-V_HG), and 3' primers specific for the J_H1, J_H2, J_H3 and J_H4 gene segments (see Methods).

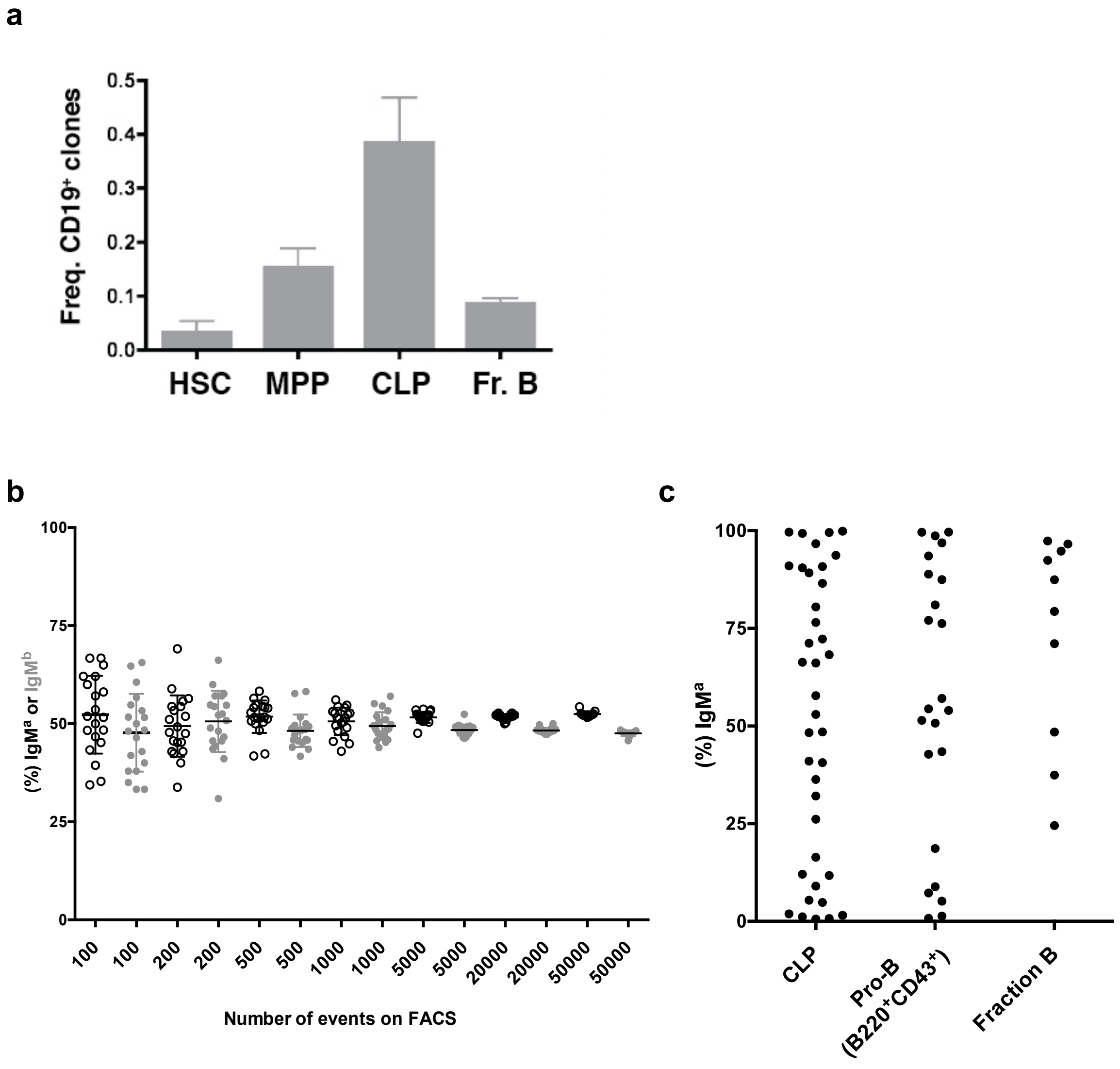

Supplementary Figure 4. *In vitro* B-cell differentiation from B-cell precursors (related to Methods, Figs. 4 and 5). (a) Mean frequency of $CD19^+$ clones in 96 well plates seeded with single-cell sorted HSC (N=384 seeded wells), MPP (N=384 seeded wells), CLP (N=267 seeded wells) or pro-B cells (Fraction B, N=384 seeded wells). The results shown are from a single experiment. (b) Estimation of the IgM^a percentage dispersion as a function of the number of acquired IgM+ cells sampled from a polyclonal population. (c) Distribution of IgM^a expression in clones derived from CLP, $B220^+CD43^+$ pro-B cells, and the fraction B (Fr.B) subset of pro-B cells. Results are from independent but identical assays.

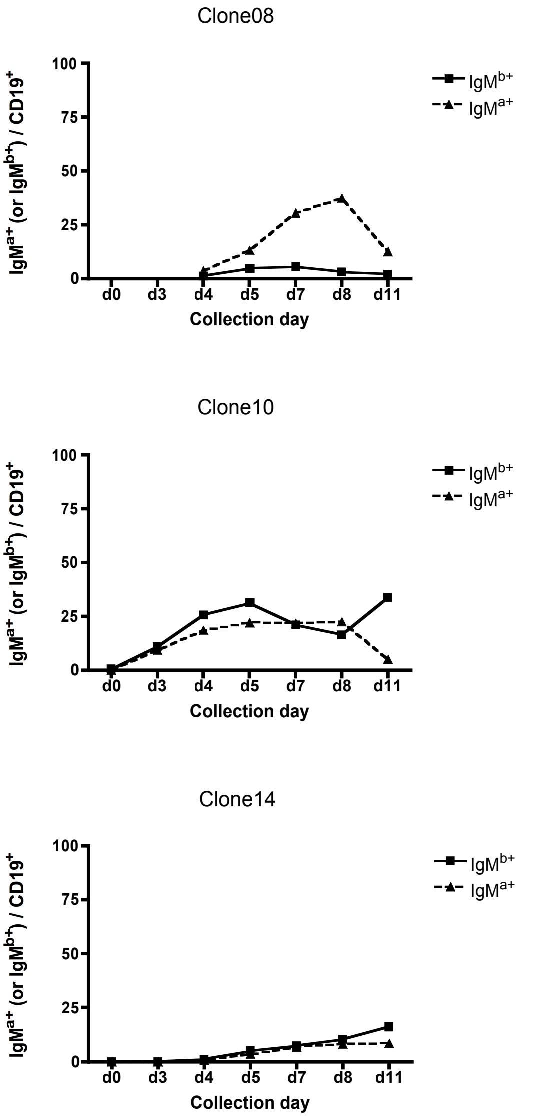

Supplementary Figure 5. Preliminary data on the IgM^a and IgM^b percentages (within the $CD19^+$) in the clones at different time points. These data were not supportive of a model in which *Igh*^{a} and *Igh*^{b} rearrange at different time points within each clone and the IgM^+ cells have a limited lifespan, which, over time, would have produced two waves of IgM^+ cells, each expressing a different allele. Since additional experimental data (see manuscript) obtained in parallel were also not consistent with this "two-wave model", the collection of data for the (IgM^a or IgM^b)/($CD19^+$) expression at different timepoints was abandoned. Data are from a single experiment; d0 is the starting day of cell culture sampling.

a

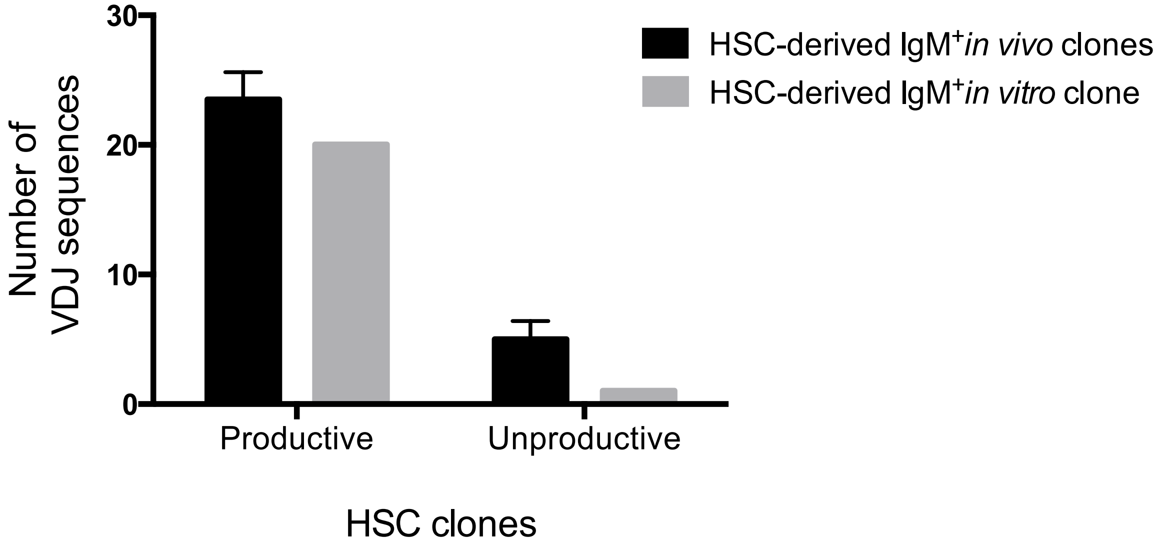
30
20
10
0
Number of VDJ sequences
HSC-derived IgM+ in vivo clones
HSC-derived IgM+ in vitro clone
Productive
Unproductive
HSC clones

b

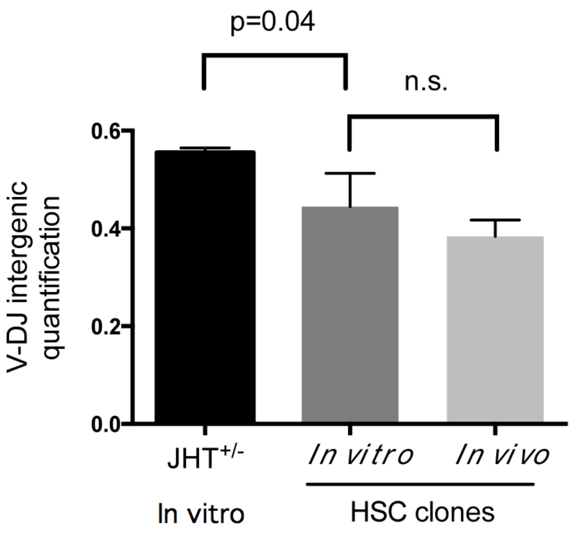
p=0.04
n.s.
0.6
0.4
0.2
0.0
V-DJ intergenic quantification
JHT+/-
In vitro
In vitro
In vivo
HSC clones

Supplementary Figure 6. Both *in vivo* and *in vitro* HSC-derived B-cell clones can rearrange a second allele after the first non-productive rearrangement, although *in vitro* the second rearrangement is less frequent (related to Figs. 4 and 5). IgM^a and IgM^b cells from HSC-derived B cell clones were sorted and DNA extracted. (a) DNA was amplified with 5' primers specific for the J558 and 7183 V_H families, and a 3' primer specific for the J_H1 gene segment. The two bands were cloned for each sample, and 8 colonies were sequenced per band in a total of 32 colonies screened per sample. We analyzed two different *in vivo*-differentiated clones and one *in vitro*-differentiated clone. The Y-axis shows the frequency of unique sequences. (b) The amount of V_H-D_H intergenic fragment was quantified in the same samples and IgM^a and IgM^b results were pooled in the same bar. The graphs show p-values from an unpaired t-test.

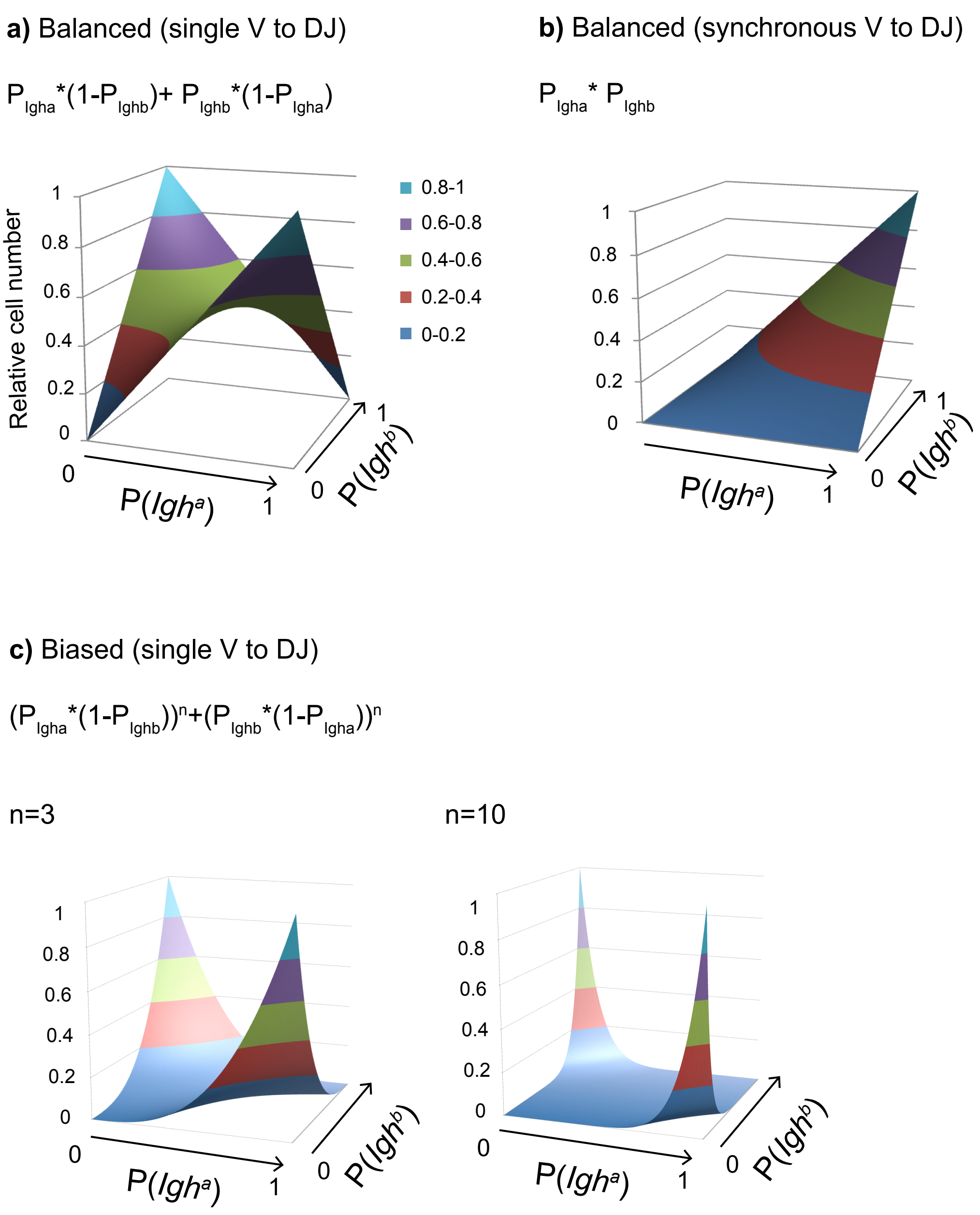

Supplementary Figure 7. Graphical representation assuming a strictly independent rearrangement of each allele predicts a majority of cells rearranging either of the alleles, a minority of cells with synchronous rearrangements and a minority of cells with biased rearrangements. The vertical axis shows the generation of pre-B cells under different combinations of P*Igh*^{a} and P*Igh*^{b}, which are the probabilities of rearranging *Igh*^{a} and *Igh*^{b}, respectively, in the time window defined by the feedback

mechanism. (a) Cells rearranging either *Igh*^{a} or *Igh*^{b} (balanced single rearrangement) predominate for most of the pairs of probabilities considered. (b) Synchronous rearrangements (i.e., the second rearrangement takes place before the feedback mechanism operates) occur in cells where both alleles have a high probability of undergoing rearrangement. (c) Populations of cells with biased rearrangements occur when there are extreme differences in the probability of rearrangement of each allele (the simulation was generated for 3 and 10 cells undergoing rearrangement in the same allele). Clonal dominance may increase the frequency of cells with biased rearrangements, particularly if the founder cell has extreme differences in the probabilities of rearranging the alleles and this property has a stable component that is transmitted to the daughter cells. In polyclonal populations the frequency of cells with biases is set by the initial frequency of founder cells with extreme differences in P*Igh*^{a} and PI *Igh*^{b}. Coupling the rearrangement probabilities per cell, a reasonable assumption given that the two alleles share the same nucleus, would decrease the frequency of cells with extreme differences in P*Igh*^{a} and P*Igh*^{b}.

Supplementary Tables

Supplementary Table 1. Antibodies and cell dyes used in flow cytometry assays (related to Methods).

Antibody[a] or Dye	Clone – Host isotype	Supplier	Conjugate, catalogue number, dilution[b] (when applicable)	Staining panel
Anti-CD16/32	2.4G2 – IgG2b	In house	1:200	FcBlock, all stainings
Anti-CD45R/B220	RA3 6B2 - Rat IGg2a	In house	(Biotin, 1:800; A488, 1:400; PE, 1:400; A647, 1:600)	Lineage cocktail and B cell progenitor detection
Anti-CD19	1D3 - Rat IGg2a	In house; eBioscience	(Biotin, 1:3200; PEcy7, cat.25-0193-82, 1:200)	Lineage cocktail and B cell detection
Anti-CD11b/Mac-1	M1/70 - Rat IGg2b	In house	(Biotin, 1:1600[c], FITC, 1:1600)	Lineage cocktail, Macrophage detection
Anti-Ly6G/GR-1	RB6-8C5 – Rat IGg2b	BD Pharmingen	(Biotin, cat.553125, 1:1600[d])	Lineage cocktail, Granulocyte detection
Anti-TER-119 Erythroid cells (Ly-76)	TER-119 – Rat IGg2b	BD Pharmingen	(Biotin, Cat.553672, 1:800)	Lineage cocktail
Anti-CD3	145.2C11 AH – IgG Group1	In house	(Biotin, 1:400)	Lineage cocktail, T cell detection

Anti-CD49b	DX5 – Rat IgM	BD Pharmingen	(Biotin, Cat.553856, 1:1600)	Lineage cocktail
Anti-CD45	30F11 - Rat IGg2b,k	BioLegend	(PE, Cat.103106, 1:1600)	Side population detection
Anti-CD150	mShad150 – Rat IgG2b	eBioscience	(PE, Cat.12-1502-82)	BM, HSC detection within SP
Anti-CD34	RAM34 - Rat IgG2a	BD Pharmingen	(FITC, Cat.56238, 1:50)	BM, HSC detection within SP
Anti-CD117 (cKit)	2B8 – Rat IgG2b	BD Pharmingen	(PE, Cat.53355, 1:400; APC, Cat.553356, 1:400)	BM, HSC and B-cell progenitors detection
Anti-LY6A/E (Sca-1)	E13-161.7 – Rat IgG2a	BD Pharmingen; BioLegend	(FITC, Cat.553335, 1:400; Pacific Blue, Cat.122520, 1:800)	BM, HSC and B-cell precursor and progenitors detection, *in vitro* assays
Anti-CD135 (Flt3/Flk2, Ly72)	A2F10 – Rat IgG2a	BD Pharmingen	(PE, Cat.553842, 1:100)	BM, B-cell precursors detection, *in vitro* assays
Anti-CD127	A7R34 – Rat IgG2a	eBioscience	(PECy7, Cat.25-1271-82, 1:100)	BM, B-cell precursor and progenitors detection, *in vitro* assays

Anti-CD45.1	A20.17 – mouse IgG2a	In house	(Pacific Blue, 1:400; PE, 1:100)	Chimeric *vs.* host cells detection
Anti-CD45.2	104 – mouse IgG2a	In house	(PE, 1:200; APC, 1:200, FITC, 1:200)	Chimeric *vs.* host cells detection
Anti-CD43	S7 - Rat IgG2a	BD Pharmingen	(PE, Cat.01605B, 1:200; FITC, Cat.553270, 1:100)	BM B-cell progenitor detection
Anti-LY-51	(6C3) BP-1 - Rat IgG2a	BioLegend	(Biotin, Cat.108304, 1:200)	BM B-cell progenitor detection
Anti-CD4	RM4-5 - Rat IgG2a	BD Pharmingen	(PE, Cat.553049, 1:400; APC-Cat.553051, 1:1600)	Thymocytes and T-cell detection and Treg staining
Anti-CD8	YTS169.4 - Rat IgG2b	In house	(Pacific Blue, 1:400, FITC, 1:800)	Thymocytes and T-cell detection
Anti-Foxp3	FJK-16s -	eBioscience	(PE, Cat.12-5773-82, 1:100)	Lymph node Treg staining
Anti-CD25	PC 61 - Rat IgG1	In house	(PE, 1:200; C5y, 1:200)	Thymocyte progenitors and Lymph node Treg staining
Anti-CD44	IM7 - Rat IgG2b	BD Pharmingen	(FITC, Cat.553133, 1:400)	Thymocyte progenitors
Anti-Ly6d	49-H4 –	BD	(FITC,	BM B-cell

	Rat IgG2c	Pharmingen	Cat.561148, 1:100)	progenitor detection
Anti-IgM total	R33.24.12 – Rat IgG	In house	(FITC, 1:400, A647, 1:3200)	B-cell sorting, *ex vivo* assays
Anti-IgM[a]	DS1 – Mouse IgG1	BD Pharmingen	(PE, Cat.553517, 1:800)	B-cell sorting and allele-specific detection, *ex vivo* and *in vitro* assays
Anti-IgM[a]	RS-31 – Mouse IgG1	In house	(A647, 1:400)	B-cell sorting and allele-specific detection, *ex vivo* and *in vitro* assays
Anti-IgM[b]	AF6-78 – Mouse IgG1	BD Pharmingen	(PE, Cat.553521, 1:800)	B-cell sorting and allele-specific detection, *ex vivo* and *in vitro* assays
Anti-IgM[b]	MB-86 – Mouse IgG1	In house	(FITC, 1:200; Cy5, 1:200)	B-cell sorting and allele-specific detection, *ex vivo* and *in vitro* assays
Anti-Ig, κ light chain	187.1 – Rat IgG1	BD Pharmingen	(PE, Cat.559940, 1:1600)	B-cell *Ig-light chain* allele-specific detection
Anti-Human-Igk	G20-193 – Mouse IgG1	BD Pharmingen	(FITC, Cat.555791, 1:50)	B-cell *Ig-light chain* allele-specific detection

Rhodamine 123	-	Invitrogen	Cat.R-302	Side population detection
Hoechst 33342	-	Invitrogen	Cat.H3570	Side population detection
Propidium Iodide	-	Invitrogen	Cat.P3566	Viability dye

[a]Anti-Mouse antibodies, except when specified as “anti-Human”; [b]all “in house” antibodies were prepared at a concentration of 2mg/mL, conjugated with the indicated fluorophore with varying and undetermined efficiency, purified, all with the same final volumes, titrated with the appropriated mouse cells and used at a final dilution as indicated; the purchased antibodies were at the concentration indicated by catalog number, and dilution was also determined empirically by titration; [c]Anti-Mac-1 biotin-conjugated antibody, when used in the lineage cocktail, was diluted even further, at a final dilution of 1:3200; [d]likewise, the anti-GR-1 biotin-conjugated antibody when used in the lineage cocktail, was used at a final dilution of 1:6400).

Suppementary Table 2. Ratios of IgM^a/IgM^b expressing B-cells in reconstituted animals (retaled to Fig. 2).

**Graft age (M)**	**Injected**	**Sample**	**CD19$^+$/IgM^{b+}**	**CD19$^+$/IgM^{a+}**	**IgM^a/IgM^b**
2.7	1 cell	#4.1_2.7M	39.7	39.5	1.0
2.7	1 cell	#4.2_2.7M	39.6	38.7	1.0
3.5	1 cell	#5.1_3.5M	45.3	40.8	0.9
3.5	1 cell	#5.2_3.5M	40.0	42.0	1.1
4.5	1 cell	#4.3_4.5M	45.7	43.4	0.9
4.5	1 cell	#4.5_4.5M	41.0	39.5	1.1
6.9	1 cell	#5.4_6.9M	34.6	36.6	1.1
3.7	1 cell	#8.1_3.7M	34.4	34.4	1.0
4.5	6 cell	#c4.4_4.5M	39.1	44.5	1.1
3.7	<50 cells	#c8.49_3.7M	33.9	40.5	1.2
3.7	50 cells	#c8.50_3.7M	33.2	39.5	1.2
2.2	50 cells	#c4.6_2.2M	35.4	36.0	1.0
2.7	1000 cells	#c5.1000_2.7M	40.1	41.2	1.0
NA	NA	Donor#245	38.7	47.9	1.2
NA	NA	Donor#4129	38.9	43.9	1.1
NA	NA	Donor#4131	39.8	45.1	1.1
NA	NA	Donor#4132	38.7	43.6	1.1

Supplementary Table 3 In clone Xp29, the non-expressed allele is unique and non-productive (related to Fig. 5).

ID[a]	V_HDJ_H (CDR3) / DJ_H junction	V_H	D_H	RF	J_H	A	F	R
	**Xp29, IgM$^-$**							
	V_HDJ_H							
1.1.	tgt **acc ggg** gat ggt tac tac tcc **tcc t**gg ttt gct tac tgg	X24.1pg.45	SP2.9	1	3	b	p	
1.2.	tgt gca aga **c**at gat ggt tac tac tcc **tcc t**gg ttt gct tac tgg	X24.1pg.45	SP2.9	1	3	b	p	
1.3.	tgt **t**at ggt tac tac tcc **tcc t**gg ttt gct tac tgg	J558.5A	SP2.9	1	3	b	p	
1.4.	tgt gca aga **gg**t ggt tac tac tcc **tcc t**gg ttt gct tac tgg	J558.6	SP2.9	1	3	b	p	
1.5.	tgt gca aga **ac**t ggt tac tac tcc **tcc t**gg ttt gct tac tgg	V186.2	SP2.9	1	3	b	p	
1.6.	tgt gca aga **tcg gga ga**t tac tac tcc **tcc t**gg ttt gct tac tgg	J558.78.182	SP2.9	1	3	b	p	
1.7.	tgt gcc aga aa**t agc cat agg gga** ggt tac tac tcc **tcc t**gg ttt gct tac tgg	Q52.a2.4	SP2.9	1	3	b	p	
1.8.	tgt gcc aga a**cc ga**t gat ggt tac tac tcc **tcc t**gg ttt gct tac tgg	Q52.2.4	SP2.9	1	3	b	p	
1.9.	tgt gcc aga **gac gg**t ggt tac tac tcc **tcc t**gg ttt gct tac tgg	Q52.a9.26	SP2.9	1	3	b	p	2
1.10.	tgt gca aga g**t**t gat ggt tac tac tcc **tcc t**gg ttt gct tac tgg	36-60.6.70	SP2.9	1	3	b	p	
1.11.	tgt gca aga g**gg ac**t gat ggt tac tac tcc **tcc t**gg ttt gct tac tgg	36-60.6.70	SP2.9	1	3	b	p	2
1.12.	tgt gca aga gag gct ggt tac tac tcc **tcc t**gg ttt gct tac tgg	36-60.6.70	SP2.9	1	3	b	p	
1.13.	tgt acc agg gat ggt tac tac tcc **tcc t**gg ttt gct tac tgg	J606.4.82	SP2.9	1	3	b	p	2
1.14.	tgc aca g**c**t ggt tac tac tcc **tcc t**gg ttt gct tac tgg	J606.1.79	SP2.9	1	3	b	p	
1.15.	tgt acc **ctc** gat ggt tac tac tcc **tcc t**gg ttt gct tac tgg	J606.4.82	SP2.9	1	3	b	p	
2.	tgt gca aga **ccc ctt gcc acg ggg g**ag *tag* c**gg gga tta tg. .**at tac tat gct atg gac tac tgg	81X	FL16.1	3	4	?	np	12
	DJ_H							
1.	c tat gat ggt tac tac tcc **tcc t**gg ttt gct tac tgg	-	SP2.9	1	3	b		4

[a] The sorted IgM$^-$ fraction of the Xp29 clone analysis is shown. Only two different DJ_H joints were detected (in sequence no. **1.** and **2.**). The DJ_H joint of sequence no **1.** is of the productive allotype (*Igh*^{b}), and is present both as a non-V_H rearranged DJ_H sequence and within diverse $V_HDJ_H^+$ rearrangements (**1.1.** – **1.13.**). The other DJ_H joint was detected in a unique sequence from the same $V_HDJ_H^-$ rearrangement. This profile suggests that the cell originally cloned had first rearranged the *Igh*^{a} allele, non-productively, and kept the *Igh*^{b} allele in the non-V_H rearranged DJ_H configuration, before or shortly after entering the culture, as a single cell, thus explaining the strong bias observed in the expressed alleles after proliferation in culture (plot Xp29 in Fig. 5c). The amount of the V_H-D_H intergenic fragment quantified was close to zero (Fig. 5a), which supports this interpretation.

Supplementary Table 4 In clone #5.3, the first rearrangement (D_H to J_H) in the *Igh*[a] allele generates a stop codon that is compatible with the allelic skewing observed (related to Fig. 5).

ID[a]	V_HDJ_H (CDR3) / DJ_H junction	V_H	D_H	RF	J_H	A	F	R
	**#5.3, IgM[b+]**							
	V_HDJ_H							
1.1.	tgt gca aga **tcg ggg g** gg gac **ggg gtg tac** tac ttt gac tac tgg	J558.67.166	Q52	3	2	b	p	
1.2.	tgt gca aga **ct**c tgg gac **ggg gtg tac** tac ttt gac tac tgg	J558.67.166	Q52	3	2	b	p	
1.3.	tgt aca aga **tcg cgg** aac tgg gac **ggg gtg tac** tac ttt gac tac tgg	J558.3.90	Q52	3	2	b	p	
1.4.	tgt gca aga **gg**c tgg gac **ggg gtg tac** tac ttt gac tac tgg	J558.80.186	Q52	3	2	b	p	
1.5.	tgt gca aga **aaa ggg aac t**[g]**g ggc** c **gg ggt gta c..** tac ttt gac tac tgg	J558.67.166	Q52	3	2	b	np	
1.6.	tgt gca aga **ctc aa**c tgg gac **ggg gtg tac** tac ttt gac tac tgg	V304	Q52	3	2	b	p	
1.7.	tgt gcc aaa **tgg gg**g gac **ggg gtg tac** tac ttt gac tac tgg	Q52.a3.8	Q52	3	2	b	p	3
1.8.	tgt gcc aga aa**t cct ccg** ggg gac **ggg gtg tac** tac ttt gac tac tgg	Q52.a2.4	Q52	3	2	b	p	
1.9.	tgt gcc aga **ggg g**gg gac **ggg gtg tac** [c]ac ttt gac tac [c]gg	Q52.2.4	Q52	3	2	b	p	
1.10.	tgt gcc aaa **gag gga gg**c tgg gac **ggg gtg tac** tac ttt gac tac tgg	Q52.a3.8	Q52	3	2	b	p	2
1.11.	tgt gc**a aga tgg g**gg gac **ggg gtg tac** tac ttt gac tac tgg	36-60.8.74	Q52	3	2	b	p	3
1.12.	tgt gca aga **gag gg**c tgg gac **ggg gtg tac** tac ttt gac tac tgg	36-60.6.70	Q52	3	2	b	p	2
1.13.	tgt gca aga ga**t gct ccc aac t**gg ac**g ggg tgt ac**. tac ttt gac tac tgg	36-60.6.70	Q52	3	2	b	np	
1.14.	tgt gca aga **gat c**ct aac tgg gac **ggg gtg tac** tac ttt gac tac tgg	7183.21b	Q52	3	2	b	p	
1.15.	tgt gca aga **caa cgg** aac tgg gac **ggg gtg tac** tac ttt gac tac tgg	7183.a7.10	Q52	3	2	b	p	
1.16.	tgt acc agg **gct aac tgt** gac **ggg gtg tac** tac ttt gac tac tgg	J606.4.82	Q52	3	2	b	p	
1.17.	tgc aca **tcc ggg gtg tac** tac ttt gac tac tgg	J606.1.79	?	-	2	b	p	2
2.	tgt gca aga gat att act acg gta **cc**g ttt gct tac tgg	S107.1.42	FL16.1	2	3	b	p	
3.	tgt gca aga **cgg gag gta ggg cc**c tat gat tac gac **ct**c tgg ttt gct tac tgg	J558.6.96	SP2.2	1	3	b	p	
4.	tgt gca aga **ggg ggg gct tt**c tat gct atg gac tac tgg	J558.39.129	?	-	4	?	p	3
5.	tgt gcc t**ct** atg att acg **ctc cgg gcg** gct atg gac tac tgg	36-60.6.70	SP2.2	2	4	?	p	2
6.1.	tgt gcc aga aa**g agg** tac tac ggt agt agc tat gg. ttt gct tac tgg	Q52.a8.22	FL16.1	3	3	a	np	
6.2.	tgt gcc aga **cta** tta cta cgg *tag tag* cta tgg ttt gct tac tgg	Q52.a8.22	FL16.1	3	3	a	np	
6.3.	tgt gca aga **cgg ggg** tta cta cgg *tag tag* cta tgg ttt gct tac tgg	36-60.a2.90	FL16.1	3	3	a	np	2
6.4.	tgt tta tta ctg tgc aat tac tac ggt agt agc tat gg. ttt gct tac tgg	J558.52	FL16.1	3	3	a	np	
6.5.	tgt aca aga aga cta cgg *tag tag* cta tgg ttt gct tac tgg	J558.35	FL16.1	3	3	a	np	
6.6.	tgt gca agt ccc cgg *tag tag* cta tgg ttt gct tac tgg	J558.f	FL16.1	3	3	a	np	
6.7.	tgt aca aga **tct** tta cta cgg *tag tag* cta tgg ttt gct tac tgg	J558.37	FL16.1	3	3	a	np	
	DJ_H							
6.	t tta tta cta cgg *tag tag* cta tgg ttt gct tac tgg	-	FL16.1	3	3	a	stp	4
	**#5.3, IgM[a+]**							
	V_HDJ_H							
6.8.	tgt gca a**gg ggg** cta tgg ttt gct tac tgg	J558.47	FL16.1	3	3	a	p	2
7.1.	tgt gca a**gg gaa** gct tac tgg	H13-3	?	-	3	a	p	2

[a] At least 18 different $V_HDJ_H^+$ rearrangements were present; 15 of these share the same *DQ52-J_H2* junction (within **1.1.** – **1.17.** sequences) and are from the *Igh*[b] allele. The identified *Igh*[a] sequences of the IgM[b] DNA (sequences **6.1.-6.7.**) are non-productive, all sharing the same *DFL16.1-J_H3* junction, which contains a stop codon in the *DFL16.1* fragment. A unique DJ_H band was present in the IgM[b] fraction of the clone as a *DFL16.1-J_H3* product. This band was sequenced and all the traces were shown to contain a single rearrangement (sequence **6.**), which shares the same *DFL16.1-J_H3* joint as the non-productive sequences. This last observation is relevant to the conclusion that, despite the phenotypic silencing of the *Igh*[a] allele, in the clone #5.3 both alleles are rearranging.

Supplementary Table 5 In clone #4.2, the skewing results from a scenario similar to that of clone #5.3: the D_H to J_H rearrangement deletes the invariant JH5' Trp codon, possibly preventing proper protein expression (related to Fig. 5).

ID[a]	V_HDJ_H (CDR3) / DJ_H junction	V_H	D_H	RF	J_H	A[b]	F	R
	**#4.2, IgM[a+]**							
	V_HDJ_H							
1.1.	tgt ac**a aga cgg gg**t atg att acg ac**g ggg** tat gct atg gac tac tgg	J558.35	SP2.2	2	4	a	p	
1.2.	tgt aca a**ta ggg ggg g**tg att acg ac**g ggg** tat gct atg gac tac tgg	J558.33	SP2.2	2	4	a	p	
1.3.	tgt gca aga **tcc ccc cc**t act atg att acg ac**g ggg** tat gct atg gac tac tgg	J558.19	SP2.2	2	4	a	p	
1.4.	tgt gcc aga a**ga** atg att acg ac**g ggg** tat gct atg gac tac tgg	Q52.a2.4	SP2.2	2	4	a	p	
1.5.	tgt gcc aga aa**c ag**t atg att acg ac**g ggg** tat gct atg gac tac tgg	Q52.a8.22	SP2.2	2	4	a	p	
1.6.	tgt gcc aaa **gtg gg**g att acg ac**g ggg** tat gct atg gac tac tgg	Q52.a3.8	SP2.2	2	4	a	p	
1.7.	tgt gcc aga ca**g ggg gtc tct atg act atg** att acg ac**g ggg** tat gct atg gac tac tgg	Q52.a15.42	SP2.2	2	4	a	p	
1.8.	tgt gcc aga g**gc ga**g att acg ac**g ggg** tat gct atg gac tac tgg	Q52.a27.79	SP2.2	2	4	a	p	
1.9.	tgt gca aga **aa**t atg att acg ac**g ggg** tat gct atg gac tac tgg	X24.a2.89	SP2.2	2	4	a	p	
1.10.	tgt gca aga c**gg gg**g att acg ac**g ggg** tat gct atg gac tac tgg	X24.a1.84	SP2.2	2	4	a	p	
1.11.	tgt gca aga c**ga g**tg att acg ac**g ggg** tat gct atg gac tac tgg	X24.a1.84	SP2.2	2	4	a	p	
1.12.	tgt gca aga cc**c cta** tct act atg att acg ac**g ggg** tat gct atg gac tac tgg	X24.a1.84	SP2.2	2	4	a	p	
1.13.	tgt gca aga **tcg** atg att acg ac**g ggg** tat gct atg gac tac tgg	36-60.a2.90	SP2.2	2	4	a	p	
1.14.	tgt gca aga **tca gg**t atg att acg ac**g ggg** tat gct atg gac tac tgg	36-60.a2.90	SP2.2	2	4	a	p	2
1.15.	tgt gca aga t**cc** atg att acg ac**g ggg** tat gct atg gac tac tgg	36-60.a9.121#	SP2.2	2	4	a	p	
1.16.	tgt gca aga ca**t ggg** atg att acg ac**g ggg** tat gct atg gac tac tgg	7183.a15.24	SP2.2	2	4	a	p	
1.17.	tgt gca aga **gag aa**g ctt acg ac**g ggg** tat gct atg gac tac tgg	7183.a47.76	SP2.2	2	4	a	p	
1.18.	tgt gca ag**g g**tt acg ac**g ggg** tat gct atg gac tac tgg	7183.a13.20	SP2.2	2	4	a	p	
1.19.	tgt gca aga g**ag gag gaa** tct act atg att acg ac**g ggg** tat gct atg gac tac tgg	7183.a37.59	SP2.2	2	4	a	p	
1.20.	tgt gca aga ga**g ggc ct**t atg att acg ac**g ggg** tat gct atg gac tac tgg	D6.96	SP2.2	2	4	a	p	
1.21.	tgt **act** atg att acg ac**g ggg** tat gct atg gac tac tgg	J606.4.82	SP2.2	2	4	a	p	
1.22.	tgt acc agg **agc gaa** tct act atg att acg ac**g ggg** tat gct atg gac tac tgg	J606.4.82	SP2.2	2	4	a	p	
1.23.	tgt ac**t t**ct atg att acg ac**g ggg** tat gct atg gac tac tgg	J606.a6.127#	SP2.2	2	4	a	p	
1.24.	tgt ac**g ggg ggg** atg att acg ac[**a**] **ggg** tat gct atg gac tac tgg	J606.a6.127#	SP2.2	2	4	a	p	
2.1.	tgt gca **gga aga ggg cg**a cgg ta**c ccc gac ta**. .*gg	J558.5A	FL16.1	1	2	b	np	
	DJ_H							
2.	tt tat tac tac ggt a**cc ccg act a***gg		FL16.1	1	2	b		4
	**#4.2, IgM[b+]**							
	V_HDJ_H							
2.2.	tgt gca aga **ggg** tac tac ggt a**cc ccg act a***gg	J558.5A	FL16.1	1	2	b	p	5
2.3.	tgt gca aga **tcg ggg tct a**at tac tac ggt a**cc ccg act a***gg	J558.5A	FL16.1	1	2	b	p	3

[a] In this case, the D_H-to-J_H rearrangement of the *Igh*[b] allele (sequence **2.-2.3**.) resulted in the replacement of a highly conserved tryptophan (hydrophobic, non-polar, neutral, with big aromatic ring) with an arginine (polar, positively charged). This residue is the first one of the FR4 domain of the IgM molecule (J-H118, according to the IMGT nomenclature, H103 in the Kabat system). The position H118 has been described as invariant and forming the core of the V_H-V_L pairing interface [2]. It was reported as one of the conserved residues that, together with the "closely related" L98 F have been found in 99% of the antibody sequences (5300 different sequences analyzed) [3]. In concert with the molecular profile of low intergenic V_H-D_H fragment retention and the presence of at least one non-productive sequence in the IgM[+] fraction of this clone, we suggest that a structural defect is preventing the

proper assembly of the antibody to the surface of the cell, possibly by prohibiting a stable pairing with the light chain.

[b] Given the absence of sequence polymorphisms, the identification of the *Igh*[a] allele was inferred from the phenotypic data.

Supplementary Table 6 Sequencing analysis of V_HDJ_H and DJ_H joints of the IgM[b] and IgM[-] fractions of clone 13-18 does not exclude an allelic preference for the I*gh*[b] recruitment (related to Fig. 5).

ID[a]	V_HDJ_H (CDR3) / DJ_H junction	V_H	D_H	RF	J_H	A[§]	F	R
	**13-18, IgM[-]**							
	V_HDJ_H							
1.1.	tgt gca aga **tc**c tat ggt aac tac **ggg ggg ggc tg**t gct atg gac tac tgg	VMU-3.2	SP2.8	3	4	b	p	
1.2.	tgt gca **ttc ta**c tat ggt aac tac **ggg ggg ggc tg**t gct atg gac tac tgg	J558.34.124	SP2.8	3	4	b	p	
1.3.	tgt gca aga **tgg a**at ggt aac tac **ggg ggg ggc tg**t gct atg gac tac tgg	V303	SP2.8	3	4	b	p	
1.4.	tgt gc**g aag atg ggg** ggt aac tac **ggg ggg ggc tg**t gct atg gac tac tgg	J558.67.166	SP2.8	3	4	b	p	
1.5.	tgt gca aga **tg**t ggt aac tac **ggg ggg ggc tg**t gct atg gac tac tgg	7183.21b	SP2.8	3	4	b	p	
1.6.	tgt agt aga **t**c**g ggg ggg gct g.**t gct atg gac tac tgg	3609N.2.77	SP2.8	2	4	b	np	
1.7.	tgt aca aga **g**at ggt aac tac **ggg ggg ggc tg**t gct atg gac tac tgg	7183.14.25	SP2.8	3	4	b	p	
1.8.	tgt gca aga **ggg** ggt aac tac **ggg ggg ggc tg**t gct atg gac tac tgg	7183.23b	SP2.8	3	4	b	p	
1.9.	tgt gca agg **a**at ggt aac tac **ggg ggg ggc tg**t gct atg gac tac tgg	7183.20.37	SP2.8	3	4	b	p	
1.10.	tgt gca ag**c ggt c**at ggt aac tac **ggg ggg ggc tg**t gct atg gac tac tgg	36-60.6.70	SP2.8	3	4	b	p	
3.1.	tgt gc**t *tag* gga aag g**ta cta cgg *tag* t.. tgg ttt gct tac tgg	J558.52	FL16.1	3	3	a	np	5
4.1.	tgt gca aga g**gc gcc** gta ac**g gtc ct**g ttt gct tac tgg	D6.96	SP2.7	2	3	a	p	2
5.1.	tg**c c**tc att act acg g**a**. gcc tgg ttt gct tac tgg	J558.39.129	FL16.2	2	3	a	np	
2.1.	tct gag gac tct acg gtc *taa* tac ggc tac **gag ttt *tag*** tac ttt gac tac tgg	J558.m	FL16.2	1	2	a	np	2
2.2.	agt aag caa acc agg ggc ggc tac **gag ttt *tag*** tac ttt gac tac tgg	11.a2.92	FL16.2	1	2	a	np	
	DJ_H							
2.	tt cat tac tac ggc tac **gag ttt *tag*** tac ttt gac tac tgg	-	FL16.1	1	2	a	stp	4
6. [b]	t tta tta cta cgg *tag tag* cta **t**at tac tat gct atg gac tac tgg	-	FL16.1	3	4	?	stp	
7.	t tta tta cta cgg *tag tag* cta c**t**t gct atg gac tac tgg	-	FL16.1	3	4	?	stp	
8.	tt tat tac tac ggt agt agc tac **gta gg**g gac tac tgg	-	FL16.1	1	4	?	-	2
9.	tt tat tac tac ggt agt agc tac gct atg gac tac tgg	-	FL16.1	1	4	?	-	
10. [b]	tt tat tac tac ggt agt agc tac tat gct atg gac tac tgg	-	FL16.1	1	4	?	-	
11.	tt tat tac tac ggt agt agc tat gct tac tgg	-	FL16.1	1	3	a	-	
12.	t tta tta cta cgg *tag* tcc tgg ttt gct tac tgg	-	FL16.1	3	3	a	stp	
13.	t tta tta cta cgg *tag tag* **c**gg ttt gct tac tgg	-	FL16.1	3	3	a	stp	
14.	ttt att act acg gta gta gct acc tgg ttt gct tac tgg	-	FL16.1	2	3	a	-	
15.	tt tat tac tac ggt agt agc tac gcc tgg ttt gct tac tgg	-	FL16.2	1	3	a	-	4
	**13-18, IgM[b+]**							
	V_HDJ_H							
1.11.	tgt gca ag**t tg**t ggt aac tac **ggg ggg ggc tg**t gct atg gac tac tgg	7183.22b	SP2.8	3	4	b	p	6
1.12.	tgt gcc aga a**t**t ggt aac tac **ggg ggg ggc tg**t gct atg gac tac tgg	Q52.2.4	SP2.8	3	4	b	p	2
1.13.	tgt gcc aga **a**ag gat ggt aac tac **ggg ggg ggc tg**t gct atg gac tac tgg	VOx-1	SP2.8	3	4	b	p	4
1.3.	tgt gca aga **tgg a**at ggt aac tac **ggg ggg ggc tg**t gct atg gac tac tgg	J558.52.145	SP2.8	3	4	b	p	3
1.14.	tgt gca aga **ggg g**at ggt aac tac **ggg ggg ggc tg**t gct atg gac tac tgg	J558.16.106	SP2.8	3	4	b	p	1
	DJ_H							
16.	ttt att act acg gta gta g**cc cct** tgg ttt gct tac tgg	-	FL16.1	3	3	a	-	2

[a] In the IgM[-] fraction, V_H segments from both alleles were recruited for V_HDJ_H rearrangement. The *Igh[b]* V_HDJ_H rearrangements all share the same DJ_H joint, and are mainly $V_HDJ_H^+$ (productive). The *Igh[a]* V_HDJ_H rearrangements, on the other hand, are mainly non-productive and less diverse, with frequent stop codons. Stop codons

are also frequent in *Igh*^{a} DJ_H rearrangements. In the IgM^{b+} fraction, however, a unique *Igh*^{a} DJ_H junction was detected, that was not shared with any other V_HDJ_H of the *Igh*^{a} allotype and therefore, although *Igh*^{a} $V_HDJ_H^{+}$ sequences are present in the IgM^{-} fraction, one cannot exclude an allelic preference for the *Igh*^{b} recruitment for this clone, considering the scenario of a sub-clonal expansion.

[b] These two DJ_H junctions also appear as DJ_H junctions in clone Xp40. From our records, there is no reason to believe that these are not identical rearrangements resulting from independent events. For DJ_H junctions whithout N additions, this is a common event that is promoted by micro-homologies in the D_H and J_H germline sequences[4,5], which can explain at least one of the junctions found in two clones.

[§] Given the absence of sequence polymorphisms, the identification of the *Igh*^{b} allele was inferred from the phenotypic data.

Supplementary References

1. IMGT(®) tools for the nucleotide analysis of immunoglobulin (IG) and T cell receptor (TR) V-(D)-J repertoires, polymorphisms, and IG mutations: IMGT/V-QUEST and IMGT/HighV-QUEST for NGS. **882,** 569–604 (2012).

2. Chothia, C., Novotný, J., Bruccoleri, R. & Karplus, M. Domain association in immunoglobulin molecules. The packing of variable domains. *J. Mol. Biol.* **186,** 651–663 (1985).

3. Chothia, C. *et al.* Conformations of immunoglobulin hypervariable regions. *Nature* **342,** 877–883 (1989).

4. Gu, H., Förster, I. & Rajewsky, K. Sequence homologies, N sequence insertion and JH gene utilization in VHDJH joining: implications for the joining mechanism and the ontogenetic timing of Ly1 B cell and B-CLL progenitor generation. *EMBO J.* **9,** 2133–2140 (1990).

5. Atkinson, M. J. *et al.* Overusage of mouse DH gene segment, DFL16.1, is strain-dependent and determined by cis-acting elements. *Dev Immunol* **3,** 283–295 (1993).
